# Intermittent supplementation with *Akkermansia muciniphila* and galactooligosaccharides modulates Alzheimer’s disease progression, gut microbiota, and colon short-chain fatty acid profiles in mice

**DOI:** 10.3389/fnagi.2025.1617980

**Published:** 2025-06-30

**Authors:** Arnas Kunevičius, Akshay Kumar Vijaya, Alessandro Atzeni, Jonas Mingaila, Ieva Šimoliūnė, Rapolas Jamontas, Emilija Keževičiūtė, Miguel Gueimonde, Rolandas Meškys, Daiva Baltriukienė, Silvia Arboleya, Aurelijus Burokas

**Affiliations:** ^1^Department of Biological Models, Institute of Biochemistry, Life Sciences Center, Vilnius University, Vilnius, Lithuania; ^2^Department of Molecular Microbiology and Biotechnology, Institute of Biochemistry, Life Sciences Center, Vilnius University, Vilnius, Lithuania; ^3^Department of Microbiology and Biochemistry of Dairy Products, Dairy Research Institute IPLA-CSIC, Oviedo, Spain

**Keywords:** *Akkermansia*, Alzheimer’s disease, APP/PS1, gut-brain axis, short-chain fatty acids, galactooligosacharides, prebiotics

## Abstract

**Background:**

Bidirectional communication and mutual regulation between the gastrointestinal tract and the CNS is facilitated through the gut-brain axis. Recent studies have found reduced diversity of the gut microbiota in Alzheimer’s disease (AD) patients, and animal models suggest microbial involvement in amyloid beta peptide (Aβ) accumulation. Modulation of the gut microbiota by new-generation probiotics represents a novel treatment strategy to alleviate the symptoms and slow the progression of AD.

**Methods:**

In this study, the therapeutic effect of the probiotic *Akkermansia muciniphila* and the prebiotic galactooligosaccharides (GOS) was investigated in the APP/PS1 mouse model. After 7 months of triweekly administration, we evaluated physiological parameters, glucose metabolism, and behavioral outcomes. Additionally, we assessed gut microbiota diversity and composition, short-chain fatty acid (SCFA) concentrations in the cecum, Aβ load in the hippocampus and prefrontal cortex, and microglial abundance in the hippocampus.

**Results:**

*A. muciniphila* and GOS administration normalized fasting glucose levels, glucose metabolism, and intestinal transit time to wild-type levels. Furthermore, supplementation reduced anxiety, improved long-and short-term memory, and partially restored activity levels. It also regulated SCFA concentrations in the cecum, improved the richness of the gut microbiota, and normalized abundance of microglia in the hippocampus, indicating reduced neuroinflammation.

**Conclusion:**

These findings suggest that long-term administration of *A. muciniphila* and GOS effectively improves metabolic health and modulates symptoms of AD in the APP/PS1 mouse model.

## Introduction

Alzheimer’s disease (AD) is a progressive neurodegenerative disorder with a high social and economic impact (Singh et al., 2024). The classic characterization of AD is based on the extracellular accumulation of amyloid-β (Aβ) plaques and neurofibrillary tangles (Hardy and Allsop, 1991). The increase in insoluble Aβ molecules and the formation of neurofibrillary tangles are influenced by a number of factors, particularly, the hyperphosphorylation of the Tau protein and mutations in the α, β, γ-secretases that cleave Aβ in the extracellular space. These changes exacerbate neuroinflammation and chronic microglial activation, culminating in the loss of neurons and disruption of synaptic signaling (Kocahan and Doğan, 2017). Furthermore, soluble amyloid monomers, such as Aβ_42_, facilitate the formation of soluble oligomers, which can diffuse to the synaptic cleft and disrupt synaptic plasticity (Selkoe, 2008). The complex pathophysiology of dementia and AD in particular poses a number of challenges when it comes to understanding the underlying mechanisms and developing efficient treatment strategies.

Current AD treatments focus on disease management and maintaining quality of life rather than directly treating the underlying causes of the disease. Traditional AD treatment options include acetylcholinesterase and butyrylcholinesterase inhibitors, and NMDA receptor antagonists (Singh et al., 2024). However, the efficacy of these medications remained questionable, with mild or temporary benefits to patients’ well-being (Chang et al., 2021; Marucci et al., 2021). There is also a new generation of pro-drugs that are only bio-oxidized after crossing the blood-brain barrier and exerts the effect on the cholinergic system at lower doses and with fewer adverse gastrointestinal symptoms (Bohn et al., 2015). However, the limited number of clinical trials and the possibility of non-specific hydrolysis by the gut microbiota pose additional hurdles to the practical application of AD pro-drugs (Marucci et al., 2021). Novel therapies, such as monoclonal anti-Aβ antibody-based treatments, have shown promising results in clinical trials by reducing the pathological burden of Aβ plaques. These therapies specifically target high-molecular weight fibrillar Aβ aggregates, thereby slowing AD progression (Cummings et al., 2024). However, the potential long-term side effects of these treatments remain uncertain, with an increased risk of amyloid-related imaging abnormalities (Lacorte et al., 2022). The limited efficacy and unclear safety profile of current AD therapies emphasize the need for a new approach that could address the causes of neuronal and functional loss.

The human gut microbiota, which consist of over 1,000 unique species with vast metabolic potential, plays a crucial role in influencing the CNS in both health and disease (Jiang et al., 2017; Arnoriaga-Rodríguez et al., 2020). Increasing evidence suggests that it may also serve as a potential therapeutic target for neuropsychiatric disorders (Cryan et al., 2019; Long-Smith et al., 2020; Cai et al., 2023). The effect of gut microbiota on the brain is facilitated via bidirectional communication called the gut-brain axis. The gut microbiota exerts its effects on the CNS through humoral, immune, endocrine, and metabolic pathways (Burokas et al., 2015). Numerous studies have reported changes in the gut microbiota and reduced microbial diversity in neurodegenerative disorders (Naseribafrouei et al., 2014; De Angelis et al., 2015; Mulak and Bonaz, 2015; Megur et al., 2020). Of particular interest are bacteria that produce neuroactive compounds, such as neurotransmitters or their precursors, short-chain fatty acids (SCFA), tryptophan (Megur et al., 2023), and peptides (Skowron et al., 2022). Despite comprehensive understanding of the metabolic potential of probiotics, their direct use in the prevention or treatment of neurodegenerative disorders remains limited.

The potential of probiotics as a therapeutic or management strategy for AD is still emerging. This is primarily because the key compounds that are potent enough to reduce Aβ burden and the bacterial species that can produce these molecules in biologically significant quantities are only known to a limited extent. Nevertheless, *postmortem* studies suggest that AD patients have significantly lower GABA levels in the cortex (Lanctôt et al., 2004). A similar disease phenotype has been observed in patients with essential tremor (Kuo and Louis, 2022). Treatment with *Lactobacillus plantarum L5* in a murine model of essential tremor resulted in increased function and concentration of GABA in the cerebellum (Zhong et al., 2023). In the transgenic AD mouse model, administration of the probiotic mixture Lab4b (mixture of *Lactobacillus salivarius CUL61*, *Lactobacillus paracasei CUL08*, *Bifidobacterium bifidum CUL20*, and *Bifidobacterium animalis* subsp. *lactis CUL34*) led to an improvement in memory, a reduction in neuroinflammatory markers and increased mRNA expression of *brain-derived neurotrophic factor* (*BDNF*). Similarly, extensive probiotic cocktails utilizing 10 distinct strains were successfully used in reducing progression and cognitive decline in AD mice models (Prajapati et al., 2025). Clinical trials reported similar results after the administration of a mixture of *Lactobacillus* and *Bifidobacteria* for 12 weeks, with improved cognition, decreased level of C reactive protein, and increased insulin resistance (Akbari et al., 2016). With no significant side effects (Akbari et al., 2016), these results emphasize the potential of probiotics in managing symptoms of AD.

Although administration of classical probiotics (*Lactobacillus* and *Bifidobacterium*) shows some improvement in brain function and a reduction in inflammatory markers, next-generation probiotics (NGPs) with the ability to produce high concentrations of bioactive metabolites hold promise for more effective management of AD. Notable candidate for NGPs include: multiple members of *Bacteroides* genus (*Bacteroides thetaiotaomicron*, *Bacteroides acidifaciens*, *Bacteroides uniformis*) (Jan et al., 2024), *Akkermansia muciniphila* (Higarza et al., 2021), *Eubacterium hallii* (Almeida et al., 2020), *Blautia wexlerae* (Samulėnaitė et al., 2024) and *Roseburia intestinalis* (Heinken et al., 2014; Jan et al., 2024). In particular, the abundance of *A. muciniphila* has been recognized as an important marker of human health, with lower levels of *A. muciniphila* observed in inflammatory bowel disease, obesity, and type II diabetes (Al-Fakhrany and Elekhnawy, 2024). Oral administration of *A. muciniphila* has been reported to reduce systemic inflammation, which is a critical component in the progression of most neurodegenerative disease (Raftar et al., 2022). Furthermore, prebiotics such as galactooligosaccharides (GOS) promote the growth of probiotic bacteria and reduce neuroinflammation (Vijaya et al., 2024) and have an anxiolytic effect (Burokas et al., 2017). Inflammation is a major factor in the pathogenesis of AD (Kinney et al., 2018) and the use of next-generation probiotics could slow-down the progression and modulate symptom AD severity (Ou et al., 2020). Building on these findings, we aimed to evaluate the therapeutic potential and potential synergistic effect of intermittent administration of *A. muciniphila* and GOS in a transgenic AD mouse model. The APP/PS1 mouse line was used to investigate whether chronic administration of *A. muciniphila* in conjunction with GOS can alleviate the symptoms of AD by regulating SCFAs levels and reducing the number of microglia in the hippocampus and the Aβ burden in the brain. GOS were selected based on their well-documented synbiotic effects, including the promotion of *A. muciniphila* growth and the increase Treg cell levels (Lindenberg et al., 2021; Li et al., 2023). In addition, we evaluated the impact of the synbiotic combination of *A. muciniphila* and GOS on gut microbiota diversity, metabolic regulation, glucose metabolism, and functional markers assessed through behavioral testing.

## Materials and methods

### Animals and genotyping

All animal experiments were performed in accordance with the Directive 2010/63/EU of the European Parliament and of the Council and approved by the Lithuanian State Food and Veterinary Service (Project No G2-244). C57BL/6JRj (*n* = 8, Janvier Labs, France) B6.Cg-Tg (APPswe, PSEN1dE9)85Dbo/Mmjax, MMRRC ID: 34832 (APP/PS1) 12-week old mice (*n* = 22, The Jackson Laboratory, United States) were used for this study. APP/PS1 mice overexpress a chimeric mouse and human-mutated amyloid precursor protein (APP) with the Swedish mutation (APP695Swe) and a mutant human presenilin 1 gene with exon 9 deletion (PSEN1dE9). Genotyping of APP/PS1 mice was performed according to the official JAX protocol using the touchdown cycling protocol with 5′-GTGTGATCCATTCCATCAGC-3′, 5′-GGATCTCTGAGGGGTCCAGT-3′, 5′-ATGGTAGAGTAAGCGAGAACACG-3′ primer sequences (Supplementary Figure S1). All animals were maintained under controlled conditions in a 12-h light–dark cycle at a temperature of 22 ± 1°C and a humidity of 55 ± 3%. All animals were provided with regular chow and had ad libitum access to water. They remained under veterinary supervision for the entire duration of the experiment.

Before the experiment, the animals were divided into four groups: WT—C57BL/6JRj; APP/PS1—APPswe, PSEN1dE9 AD mice model; APP/PS1 + *A. muciniphila*—AD mice model supplemented with *A. muciniphila*; APP/PS1 + *A. muciniphila* + GOS AD mice model supplemented with *A. muciniphila* and GOS.

### Administration of *Akkermansia muciniphila* and GOS

**Figure 1:**
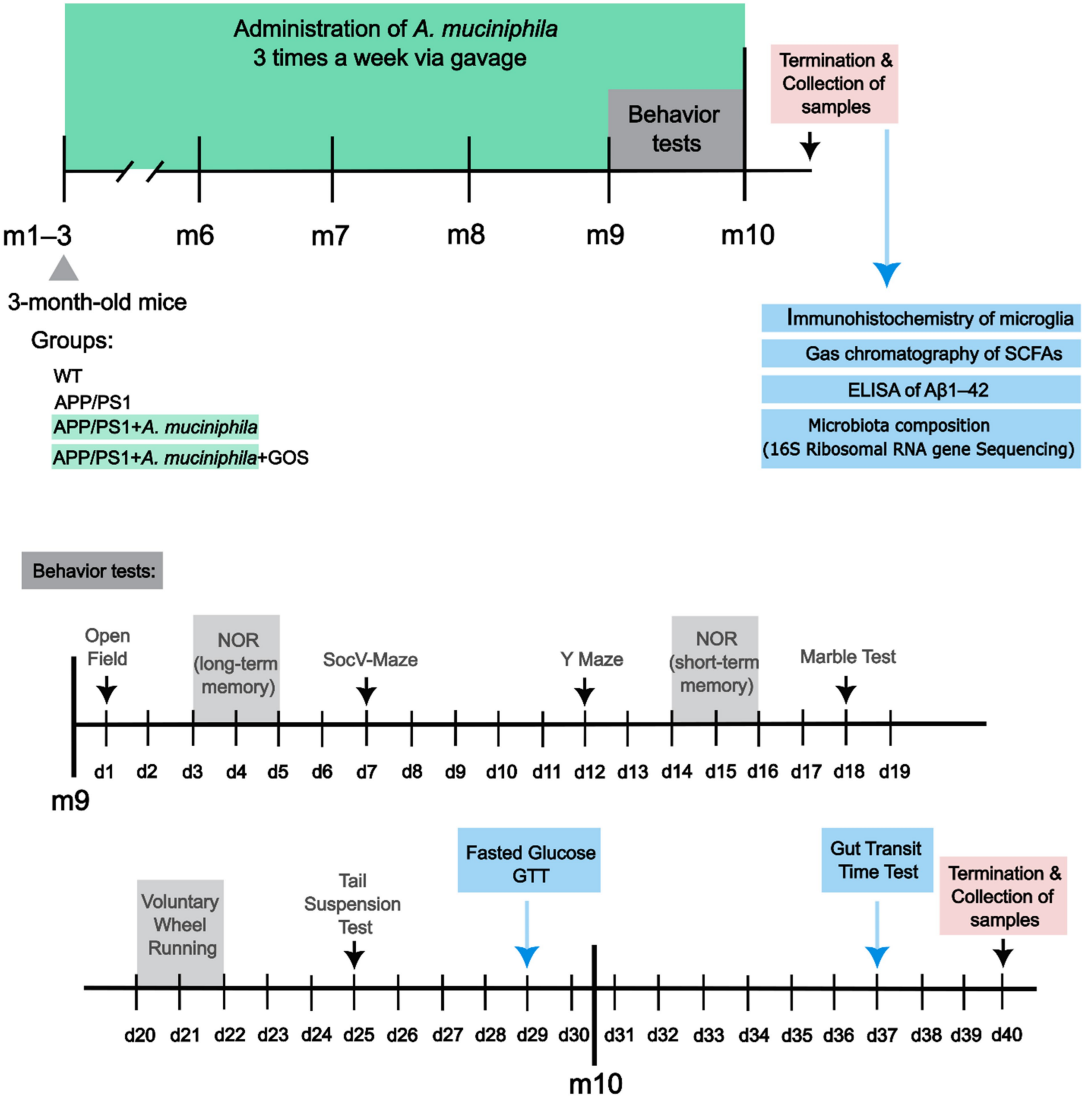
A timeline with the tests that the animals have undergone in chronological order.

All animals received same standard feed (with 3.87 kcal/g energy value, Altromin, Germany) and autoclaved water. APP/PS1 + *A. muciniphila* + GOS received water supplemented with 3% GOS (King-Prebiotics^®^, Yunfu City, Biotechnology Corporation Limited, China). The animals in the *A. muciniphila* and *A. muciniphila* + GOS groups were gavaged with the bacteria intermittently (3 times a week) for 7 months (Figure 1). After thawing on ice, *A. muciniphila* was centrifuged, diluted in sterile PBS and each animal received 100 μL of the solution containing 1 × 10^9^ CFU of the bacteria.

### Bacterial cultivation

Bacterial cultivation for animal experiments: *A. muciniphila* CIP107961 was grown in GAM medium (Nissui Pharmaceutical Co.) and incubated at 37°C in an anaerobic chamber (Mac 500; Don Whitley Scientific) under a 10% (v/v) H2, 10% (v/v) CO_2_ and 80% (v/v) N_2_ atmosphere. Twenty-four hours culture was used to inoculate (1% v/v) pre-reduced GAM broth medium, which were incubated for another 24 h. Afterward, the culture was washed and concentrated with pre-reduced PBS plus 20% (v/v) glycerol to about 1 × 10^10^ CFU/mL and, stored at −80°C until administration (Higarza et al., 2021).

Bacterial cultivation for *in vitro* experiments: *A. muciniphila* was cultivated in stationary cultures anoxically at 37°C in liquid brain heart infusion (BHI) medium (37 g/L, Carl Roth, Germany, #X916.2) supplemented with porcine stomach mucin (4 g/L, Sigma-Aldrich, United States, #M1778). An appropriate amount of GOS was added to the medium as indicated in the Results section. To prepare agar plates, 1.5% agar was added to the medium. Anaerobic manipulation of the bacteria was carried out either in a 100% N_2_ environment using the Unilab Pro glovebox (Mbraun, Germany) or by syringe using glass bottles with rubber stoppers flushed with sterile N_2_. The growth curves were determined by spectrophotometric measurements with the NanoPhotometer (C40, Implen, Germany). For the growth curves, 2 mL of the bacterial suspension were grown as stationary cultures in 2 mL test tubes in a dry heating block. The presence of *A. muciniphila* was confirmed using end-point PCR (Supplementary Figure S2) for the species-specific sequence with 5′-CAGCACGTGAAGGTGGGGAC-3′ and 5′-CCTTGCGGTTGGCTTCAGAT-3′ pair of primers adapted from Kim et al. (2021).

### Animal weight and glucose tolerance test

Experiments with animals began at 3 months of age and concluded at 10 months. The study was conducted over a span of 7 months. Glucose tolerance test (GTT) was performed to check glucose metabolism impairment as previously described (Burokas et al., 2018). During the GTT, glucose level was measured at different time points up to 2 h. The animals were not fed for 14 h before the experiment but were provided with water. After measuring basal levels of glucose (0 timepoint), animals were injected with glucose 2 g/kg intraperitoneally (i.p.), and blood glucose levels were measured at 15, 30, 45, 60 and 120 min. Five microliters of blood was collected from the snipped tail to measure glucose levels using a blood glucose meter (CONTOUR^®^NEXT GEN, United States).

### Animal behavioral experiments

Standard blinding and randomization procedures, following the PREPARE guidelines, were applied to experimental animals (Smith et al., 2018). Animal housing and cage placement were randomized at the start of the experiment. All behavior experiments were recorded and later analyzed by a researcher who was blinded to group allocation. Treatment administration, behavior testing and the behavioral recordings analysis were performed by different researchers to ensure blinding.

The battery of behavioral tests was structured according to standard practice, beginning with the least stressful and moving to increasingly long and potentially stressful experiments. Before each behavioral experiment, animals were randomized to minimize order effects. A rest period of 2 to 3 days was implemented between tests (with the exception of the GTT, after which the animals rested for 5 days) to allow animals to recover and reduce potential carryover effects. The animals were acclimatized in the experimental rooms for 1 h before behavioral experiments.

### Open field test

The animals were placed in the center of the open field arena (the walls of the maze were made of white opaque Plexiglas; 40 cm long × 40 cm wide × 30 cm high) at an illumination of 500 lux (measured at floor level in the center of the arena) for 10 min, as previously described by Kulesskaya and Voikar (2014). A video recording was carried out during the experiment. The time and number of entries were counted when the animal moved to the center of the maze, which was virtually marked as a “small central square” (10 cm × 10 cm). The videos were analyzed using the Biobserve Viewer (Biobserve GmbH, Germany) software.

### Novel object recognition test

The Novel object recognition test was conducted in a V-shaped maze, as previously reported (Busquets-Garcia et al., 2011; Burokas et al., 2014). The V-shaped maze consisted of two perpendicular arms (made of black opaque Plexiglas, dimensions of the arms: 25 cm long × 5 cm wide × 15 cm high) with dim lighting (15 lux, measured at the top of the maze). The experiment consisted of three phases: habituation, training, and testing. During habituation, the animals explored an empty maze, during the training, mice examined two identical objects and then conducted the testing phase either 3 h (for the short-term memory testing) or 24 h (for the long-term memory testing) after the training. This test is based on the spontaneous tendency of rodents to spend more time exploring a novel object than a familiar one, assigned as discrimination index (DI). The DI was calculated using the following formula: DI = (T_R_ − T _L_)/(T_R_ + T_L_), where T_R_ represented the exploration time devoted to the new object, and T_L_ represented the exploration time devoted to the old object. A DI of 0.4 and above was considered the standard for good memory, and a value below 0.2 was considered as a memory impairment.

### Y-maze test

The maze was a closed Y-shaped apparatus and was illuminated from the top (15 lux, measured at the end of the arms), as first reported by Kraeuter et al. (2019). The experimental animals were placed in a grey, opaque Plexiglas arena with 3 identical arms (40 cm long × 9 cm wide × 15 cm high). Ten-minute test sessions were performed, and the number of altering arms visited was counted. Mice were considered to have entered the maze when all four limbs of the mice were within an arm zone of the maze.

### Marble test

The marble test was performed according to the protocol of Angoa-Pérez et al. (2013). Standard cages were filled with regular unscented mouse bedding material to a depth of 7 cm and leveled evenly. Twenty unicolor glass marbles (15 mm in diameter) were carefully arranged on the bedding without pressing in five rows of four marbles. After, the animals were placed in the experimental cage, which was covered with upside-down grills. Mice were allowed to explore the marbles freely for 30 min. After each session, mice were carefully returned to their home cage, and the number of partially and completely buried marbles was evaluated. Marbles were considered buried if at least two-thirds of their surface area was covered by the bedding. After each session, marbles were washed with 70% ethanol. Animals that did not exhibit marble-burying behavior were determined to be non-responsive and were consequently excluded from subsequent analysis.

### Tail suspension test

The procedure was developed based on the protocol of Steru et al. (1985). The mice were suspended by the tail using a clip placed approximately 2 cm from the tip of the tail attached to a metal bar elevated 30 cm from the surface. The duration of immobility was recorded during a single 6-min session. Mice were considered immobile only when hanging passively. All experiments were videotaped for subsequent analysis, which was performed manually by an experimenter blinded to the experimental conditions.

### Vsoc-maze test

The Vsoc-maze test was performed in a V-shaped maze according to the protocol by Martínez-Torres et al. (2019) that consisted of two perpendicular arms ending in a metal bar-separated compartment. The experiment consisted of 3 phases that were conducted in a continuous sequence: habituation, sociability testing, and social novelty testing, with each phase lasting 5 min. During habituation, the animals explored an empty maze, while during the sociability testing, the experimental animals explored a neutral object or an unfamiliar mouse. The unfamiliar mice were of the same sex, similar age, and weight. During the social novelty testing, the neutral object was replaced by a novel, unfamiliar animal. The sociability index (SI) was calculated in the same manner as DI. SI = (T_A_ − T_O_)/(T_A_ + T_O_), where T_A_ represented the exploration time devoted to the unfamiliar animal, and T_O_ represented the exploration time devoted to an inanimate object.

### Voluntary wheel running

The animals were housed individually in standard cages with a running wheel (metal wheel diameter 12 cm), provided with their respective food, and supplemented with water. Voluntary physical activity was measured by counting the number of rotations by the running wheel during a 24-h period: 12-h day cycle (cage lighting 80–100 lux, light intensity measured at the top of the cage) and 12-h night cycle (no lighting). Physical activity was recorded using a specialized recording system with PowerLab hardware, and LabChart-8 software (ADInstruments Ltd., United Kingdom) was used to measure the number of wheel rotations.

### Whole gut transit time test

Whole gut transit time was evaluated utilizing a mixture of carmine and methylcellulose, as previously described by Golubeva et al. (2017). The animals were deprived of food overnight for 16 h prior to the experiment. A solution of 0.5% methylcellulose and 6% carmine was prepared and sterilized by autoclaving. The experimental animals were gavaged with 250 μL of the carmine solution, placed in empty cages under dim lighting, and provided with water. The cages were monitored for the appearance of red fecal pellets. The transit time was considered to be the time interval between administration of carmine and appearance of the first red pellet. A maximum period of 6 h was chosen for the experiment.

### Immunohistochemistry

The number of microglia cells was determined by immunohistochemistry of coronal brain sections fixed in 4% paraformaldehyde (PFA) for 24 h, washed in PBS, and embedded in paraffin. The brain tissue was cut in 10 μm thick slices, placed on glass slides, and stored at +4°C. Microglia immunofluorescence was performed according to the protocol described by Zaqout et al. (2020). The slices were blocked with 10% goat serum (Thermo Fisher; United States) for 1 h. Rabbit anti-Iba1 primary polyclonal antibody was used (AB_2544912, 1:1000; Thermo Fisher; United States) overnight, followed by a secondary goat anti-rabbit antibody Alexa Fluor 488 (AB_143165, 1:5000; Thermo Fisher; United States) for 1 h. Tissues were then counterstained with DAPI (5 μg/mL), and fluorescence was visualized using a bright field scanning fluorescence microscope. The scanned unprocessed images were imported into the ImageJ software. The images were converted to 8-bit, and the region of interest was marked. The background was removed, and the rolling ball radius was set to 50 pixels. The number of cells was analyzed using find maxima (prominence >30, edge maxima excluded). The cells from the DAPI channel and microglia Iba1 channels were quantified, and the percentage of Iba1 positive microglia cells was calculated.

### Cecum sample collection and processing

Cecum samples from each animal were collected split into two equal parts and kept at −80°C until processing. Before SCFA analyses, samples were weighed, diluted in PBS (1:5 w/v) and homogenized (5 min, full speed) in a stomacher (LabBlender 400). From 1 mL of the homogenate, cell free-supernatants was isolated by centrifugation (10,000 rpm, 15 min). Another part of cecum was used to extract DNA for gut microbiota analysis. The cecum was homogenized using a BeadBug^™^ 6 microtube homogenizer (Merck) for three cycles of 30 s at 300 rpm, with a 30 s rest period on ice between each cycle. After homogenization, we used Quick-DNA Fecal/Soil Microbe Miniprep Kit (Zymo, D6010) to extract microbial DNA, according to the manufacturers protocol. Concentration of isolated DNA was evaluated using a spectrophotometer.

### GC/MS-FID-based SCFA analysis

The determination of SCFA concentrations was performed by gas chromatography, as described by Higarza et al. (2021). Briefly, 250 μL of cell free-supernatants were mixed with 100 μL methanol, 50 μL internal standard solution (2-ethylbutyric 1.05 mg/mL), and 50 μL of 20% v/v formic acid. The mix was then centrifuged, and the supernatant was injected into a system composed of a 6890NGC injection module (Agilent Technologies Inc., United States) connected to a flame injection detector (FID) and a mass spectrometry detector (MS, 5973 N) (Agilent).

### Gut microbiota sequencing and analysis

Library preparation and sequencing were performed by Novogene (United Kingdom) Co., Ltd. (Cambridge, United Kingdom) using the Pacbio platform, generating 10,000 clean CCS reads per sample. 16S rRNA/18S rRNA/ITS genes of distinct regions (16SV4/16SV3/16SV3-V4/16SV4-V5, 18SV4/18SV9, ITS1/ITS2, ArcV4) were amplified used specific primer (e.g., 16SV4: 515F-806R, 18SV4: 528F-706R, 18SV9: 1380F-1510R, et al.) with the barcode. All PCR reactions were carried out with 15 μL of Phusion^®^ High-Fidelity PCR Master Mix (New England Biolabs); 0.2 μM of forward and reverse primers, and about 10 ng template DNA. Thermal cycling consisted of initial denaturation at 98°C for 1 min, followed by 30 cycles of denaturation at 98°C for 10 s, annealing at 50°C for 30 s, and elongation at 72°C for 30 s and 72°C for 5 min.

Sequencing libraries were generated using NEB Next^®^ Ultra^™^ II FS DNA PCR-free Library Prep Kit (New England Biolabs, United States, Catalog #: E7430L) following manufacturer’s recommendations and indexes were added. The library was checked with Qubit and real-time PCR for quantification and bioanalyzer for size distribution detection. Quantified libraries were pooled and sequenced on Illumina platforms, according to effective library concentration and data amount required.

Alpha diversity indices for the gut microbiota—*Chao1*, *Shannon*, and *Simpson* (Shannon, 1948; Simpson, 1949; Chao, 1987)—were computed based on the absolute amplicon sequence variant (ASV) counts, and their relationship to the study groups was assessed by linear regression. Gut motility is an important factor shaping gut microbiota (Tottey et al., 2017), thus we adjusted for whole gut intestinal transit time (in seconds) following Mingaila et al. (2023) data analysis work flow. Results with *p* < 0.05 were considered significant.

Beta diversity was assessed by calculating Euclidean distance on log-ratio transformed relative abundance genus counts (Aitchison et al., 2000), including only taxa with a relative abundance of ≥0.001 in at least 10% of samples. Using the “adonis2” function in the R *vegan* package (version 2.6-6.1) (available at https://CRAN.R-project.org/package=vegan), permutational multivariate analysis of variance (PERMANOVA) (Anderson, 2001) was conducted to assess differences in microbiota composition according to study groups. Model was adjusted for whole gut intestinal transit time and *p* < 0.05 as significant threshold.

Principal component analysis (PCA) was performed to illustrate the variation between study groups in a two-dimensional plot.

Differential abundance analysis was conducted using the *MaAsLin2* R package (17) to test the association between specific gut microbiota taxa and study groups. General linear models were applied to log-ratio transformed and genus counts prior abundance and prevalence filtering, and accounting for carmine intestinal transit time as covariate. No further filtering was applied in the model. The analysis included Benjamini–Hochberg adjusted *p*-values, focusing comparisons on the primary predictor with covariate control. Findings with Benjamini–Hochberg adjusted *p* < 0.05 were reported.

The association between study groups and the log-ratio transformed relative abundance of the *Akkermansia* genus and *A. muciniphila* was tested using linear regression, adjusting for whole gut transit time (in seconds). Results with *p* < 0.05 were considered significant.

Spearman’s correlation was performed to assess the relationship between differentially abundant taxa and a dataset containing various variables. Since the dataset included continuous variables and it was assumed that these variables were missing completely at random (MCAR), multiple imputation using predictive mean matching (PMM) was applied (Rubin, 1987). PMM was considered an appropriate approach because it preserves the distribution of the variable (Morris et al., 2014; van Buuren, 2018) and imputation is performed by matching observed values with similar patterns (Schenker and Taylor, 1996). As the variables were measured on different scales (e.g., SCFA in mM, behavioral scores, and microglia percentages), standardization was conducted to prevent bias. The correlations are filtered based on the criteria absolute correlation coefficient |*r*| > 0.5 and adjusted *p* < 0.05 to identify statistically significant and practically meaningful relationships in the data.

### ELISA assay

Quantification of Aβ_(1–42)_ levels in the soluble and insoluble fractions, was performed according to the protocol of Bracko et al. (2020). Frozen mouse brain hippocampus and prefrontal cortex tissues were homogenized in 1 mL PBS with complete protease inhibitor cocktail (Roche), 1 mM PMSF (Sigma), and 2 mM sodium orthovanadate (Sigma) using a Dounce homogenizer on ice. The homogenized tissues were vortexed and centrifuged at 14,000 g for 30 min at 4°C. The soluble fraction was removed and stored at −80°C until further analysis. The remaining pellet was dissolved in 0.4 mL of 70% formic acid, shaken, and centrifuged at 14,000 g for 30 min at 4°C to dissolve the insoluble fraction. The supernatant was removed and neutralized with 1 M Tris-base buffer at pH 9. Protein concentrations were measured using Bradford Protein Assay (Thermo Fisher Scientific, United States). The soluble fraction was diluted 4-fold and the insoluble fraction was diluted 30-fold before the ELISA assay was performed. The solid-phase sandwich ELISA (KHB3441, Thermo Fisher Scientific, United States) was performed according to the protocol provided. The Aβ_(1–42)_ concentrations were calculated by comparing the optical density of the sample with the optical density of the Aβ_(1–42)_ standard calibration curve within the same plate. The data were standardized according to the extracted total protein concentration in the both soluble and insoluble fractions.

### Statistical analysis

All grouped analysis data were presented as mean ± SEM. The data were analyzed and graphed using Prism 9 (GraphPad software), and Inkscape. Gut microbiota statistical analyses were carried out using R (version 4.4.1) and RStudio (version 2023.6.0.421). The Shapiro–Wilk test was applied to assess the normality of the data. Statistical significance was determined using Welch’s ANOVA, followed by Dunnett’s Multiple Comparisons Test, unless stated otherwise. Differences were considered significant at ^*^p ≤ 0.05, ^**^p ≤ 0.01, ^***^p ≤ 0.001.

## Results

### The influence of galactooligosacharides on the growth of *Akkermansia muciniphila*

**Figure 2:**
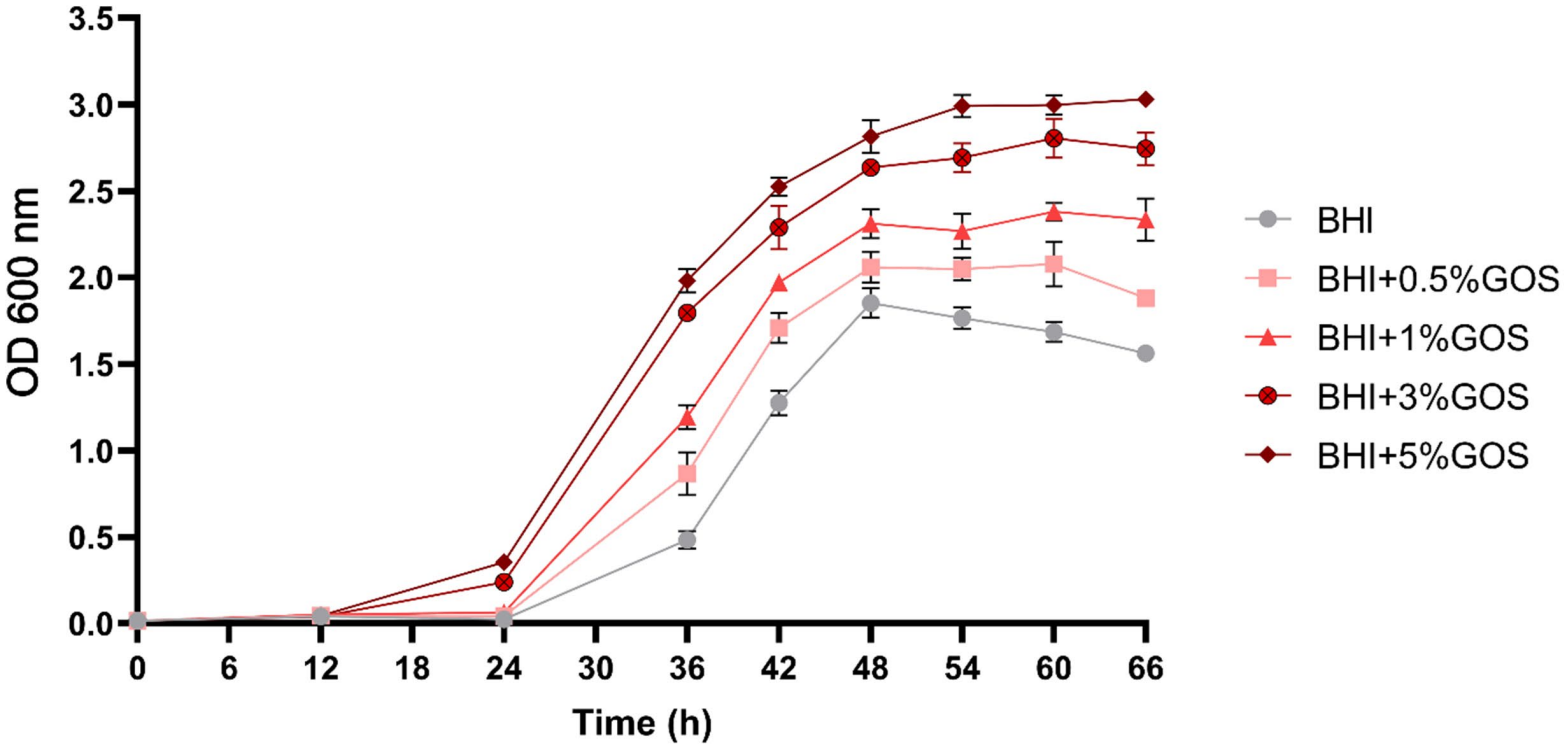
Spectrophotometric measurement of the growth *A. muciniphila* with different concentrations of GOS. *N* = 3 per group. Data are presented as mean ± SD.

To confirm the ability of GOS to improve the growth of *A. muciniphila*, the bacteria were incubated in a liquid BHI medium with different concentrations of GOS (Figure 2). We observed a concentration-dependent increase in optical density with an exponential growth stage starting after 24 h. Significant growth differences were observed between all investigated groups at all time points, beginning at 36 h. 3 and 5% GOS provided the best conditions for the growth of *A. muciniphila*. Although the growth differences between 3 and 5% GOS were statistically significant, they remained minimal. Given our study’s focus on a mild intervention, we selected the 3% GOS concentration for *in vivo* experiments.

### Physiological and behavioral changes induced by the administration of *Akkermansia muciniphila* in APP/PS1 mice

**Figure 3:**
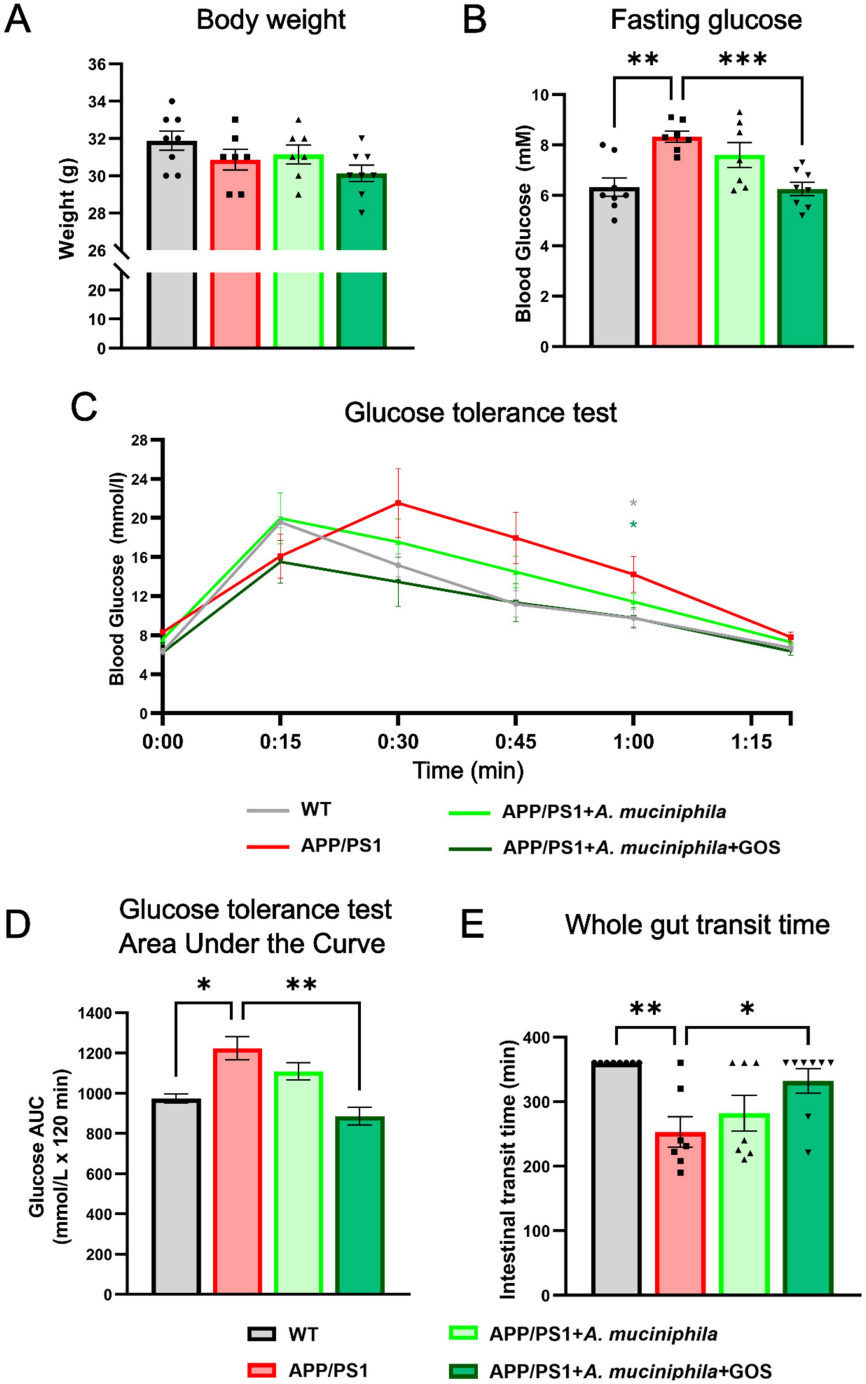
Functional markers of 10-month-old animals after 7 months of supplementation with *A. muciniphila*. **(A)** Weight measurement. **(B)** Fasting glucose assessment. **(C)** Glucose tolerance test. **(D)** Quantitative area under the curve representation of glucose tolerance test. **(E)** Whole gut transit time test. WT *N* = 8, APP/PS1 *N* = 7, APP/PS1 + *A. muciniphila N* = 7, APP/PS1 + *A. muciniphila* + GOS *N* = 8. Data are presented as mean ± SEM. Statistically significant differences are shown in comparison to the APP/PS1 group. ^*^p ≤ 0.05, ^**^p ≤ 0.01, and ^***^p ≤ 0.001.

Obesity and elevated fasting glucose levels are important risk factors for AD. Metabolic syndrome increases the level of inflammation in the body and accelerates the accumulation of Aβ may play a role in the progression of AD (Gomez et al., 2018). To evaluate whether the administration of *A. muciniphila* could improve the metabolic status of APP/PS1 mice, we measured the weight and glucose levels of animals that were fasted for 14 h. No differences in weight were observed (Figure 3A). However, APP/PS1 mice had significantly higher levels of fasting glucose which were restored to WT levels by administration *A. muciniphila* and GOS (Figure 3B). The disruption of glucose metabolism was confirmed by the GTT in which APP/PS1 animals had higher glucose levels 1 h after glucose administration (Figure 3C) and a larger area under the curve overall (Figure 3D). The combination of *A. muciniphila* and GOS was effective in restoring glucose metabolism to WT levels (Figure 3D). Furthermore, APP/PS1 animals exhibited a faster intestinal transit time which was normalized with *A. muciniphila* and GOS supplementation (Figure 3E). These data show the potential of the treatment with *A. muciniphila* and GOS to improve the metabolic state in AD patients.

**Figure 4:**
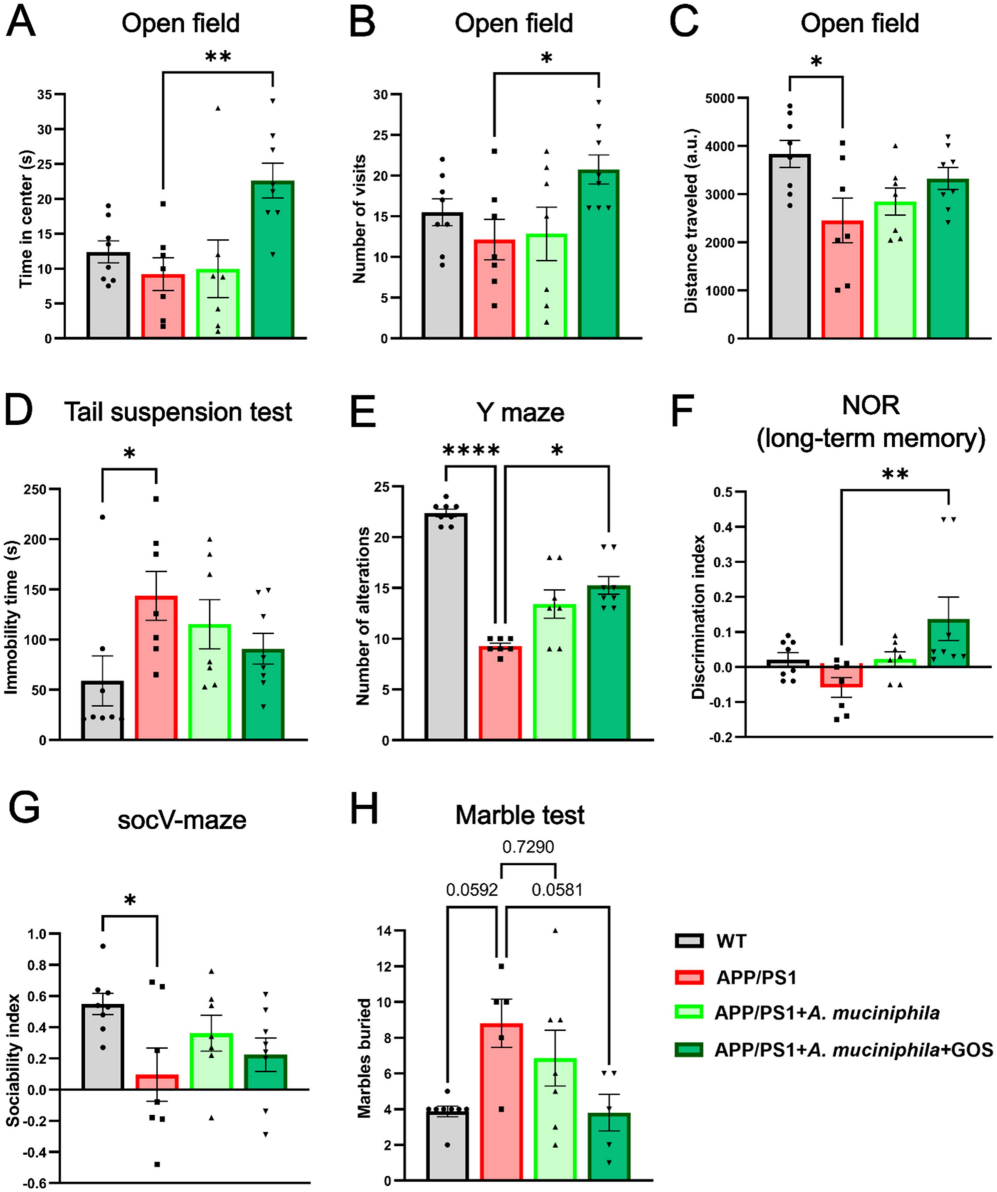
Behavior assessment of APP/PS1 animals. **(A)** Time in the center of the arena time in the open field test. **(B)** Number of visits to the center of the arena in the open field test. **(C)** Total distance traveled during total active time in the open field test. **(D)** Time of immobility in the tail-suspension test. **(E)** Number of alterations during the Y-maze test. **(F)** Long term memory assessment using the novel object recognition test. **(G)** Sociability assessment using the Vsoc-maze test. **(H)** Anxiety assessment using marble test. WT *N* = 8, APP/PS1 *N* = 7, APP/PS1 + *A. muciniphila N* = 7, APP/PS1 + *A. muciniphila* + GOS N = 8. Data are presented as mean ± SEM. ^*^p ≤ 0.05, ^**^p ≤ 0.01, and ^***^p ≤ 0.001. Statistically significant differences are shown compared to APP/PS1 group.

Lower levels of fasting glucose and slower glucose metabolism showed that the effects of *A. muciniphila* and GOS extend beyond the gut, manifesting systemically in the blood. However, it was still unclear whether the prebiotic-probiotic mixture could exert its effect on the CNS in an AD model. To investigate this, we conducted a series of behavioral tests designed to assess anxiety, activity levels, depressive behavior, memory, and sociability. Supplementation with *A. muciniphila* and GOS significantly increased both the time spent and the number of visits to the center in the open field test, indicating reduced anxiety (Figures 4A,B). Additionally, the open field test revealed a significantly lower activity in APP/PS1 animals with a trend (*p* = 0.150) towards partial recovery of activity in the *A. muciniphila* and GOS treated animals (Figure 4C). A similar trend (*p* = 0.076) was observed in the voluntary wheel running test (Supplementary Figure S3A), where APP/PS1 mice tended to show lower activity, which was fully restored to the WT level by the administration of *A. muciniphila* and GOS. Furthermore, APP/PS1 animals exhibited high immobility time in the tail-suspension test (Figure 4D), but the administration of *A. muciniphila* and GOS showed no effect towards the depression-like phenotype. Regarding the working memory, the Y-maze test revealed that APP/PS1 mice showed a dramatic decrease in the number of alterations (Figure 4E), which was partly reversed by the administration of *A. muciniphila* and GOS. The effect on memory was confirmed in the novel object recognition test with an increased discrimination index in the group supplemented with *A. muciniphila* and GOS (Figure 4F). The results of the short-term memory test supported these findings (Supplementary Figure S3B). This indicates the ability of *A. muciniphila* and GOS to prevent the memory deficits observed in the APP/PS1 AD model. APP/PS1 animals exhibited lower levels of sociability compared to WT animals and supplementation with *A. muciniphila*, either alone or in combination with GOS, did not result in a significant improvement in social behavior (Figure 4G). An increased frequency of repetitive behaviors is a common sign of anxiety in mice (Bailey and Crawley, 2009). A trend (*p* = 0.058) of an increased number of buried marbles was observed in APP/PS1 mice (Figure 4H), despite a reduction in overall activity levels (Figure 4C). Administration of *A. muciniphila* and GOS significantly reduced repetitive behavior to the WT levels (Figure 4H). Overall, treatment with *A. muciniphila* and GOS demonstrated the ability to mitigate AD-related behavioral traits in the APP/PS1 mice (summarized in Table 1).

**Table 1:** Summary table of behavioral outcomes after supplementation with *A. muciniphila* and GOS.

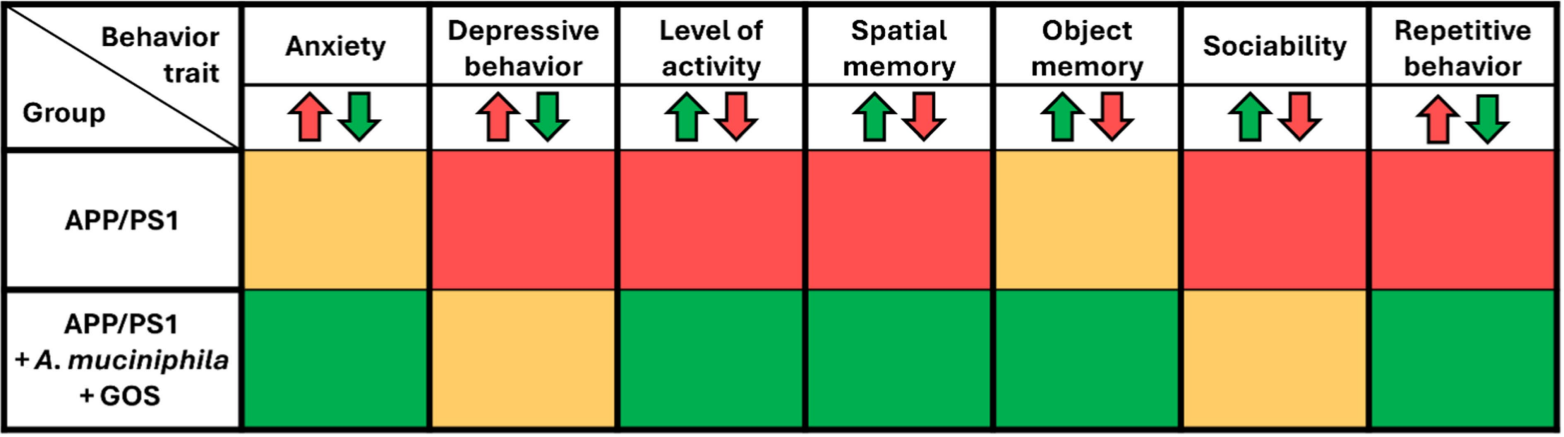

APP/PS1 compared to WT, APP/PS1 + *A. muciniphila* + GOS group compared to APP/PS1. Green—improved behavior, yellow—no changes, red—deterioration in behavioral trait.

### The effect of *Akkermansia muciniphila* and GOS supplementation on Aβ burden in the hippocampus or prefrontal cortex of APP/PS1 animals

**Figure 5:**
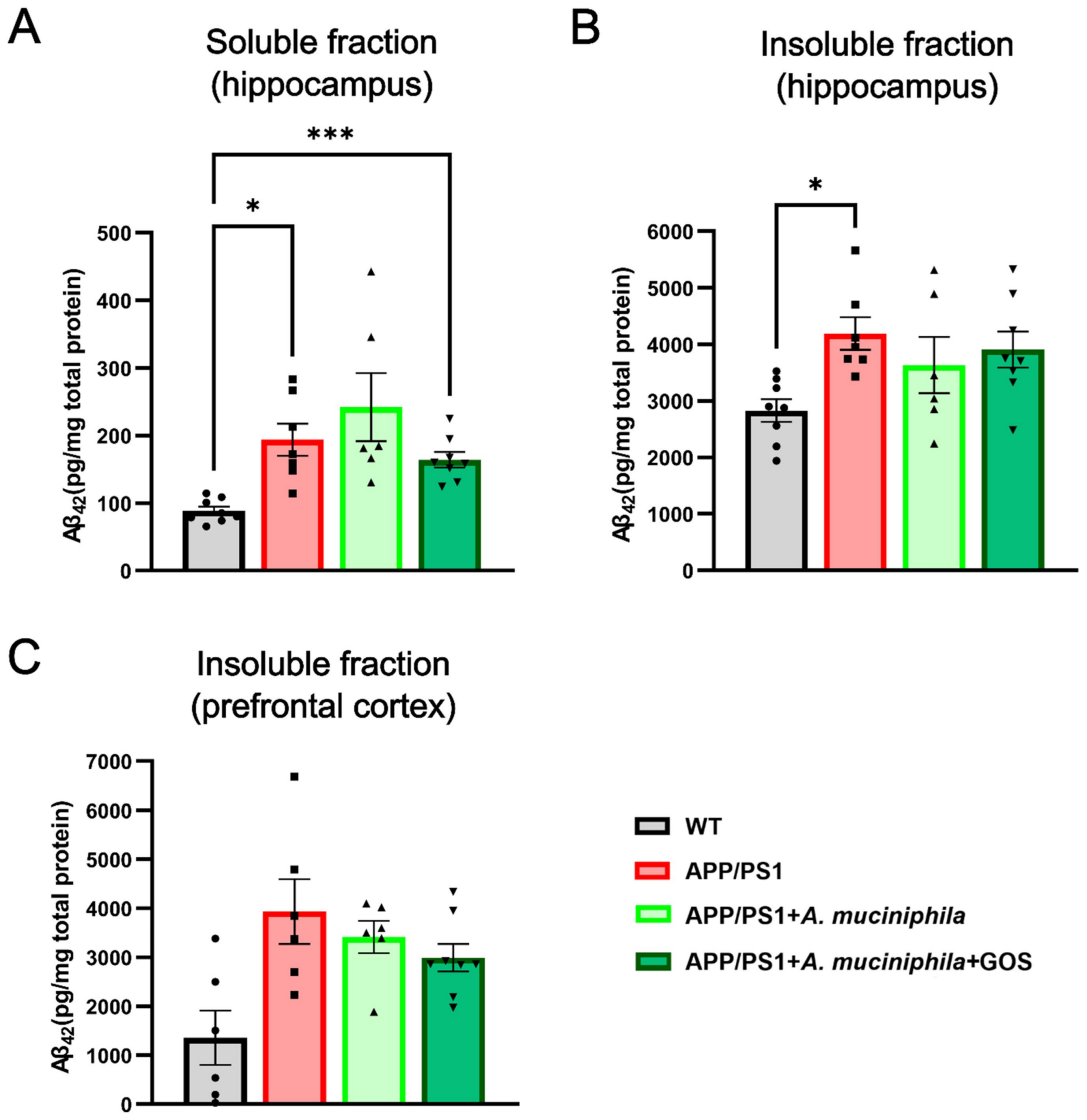
Molecular analysis of Aβ_(1–42)_ burden in WT and APP/PS1 animals. **(A)** Soluble fraction of Aβ in the hippocampus. **(B)** Insoluble fraction in the hippocampus. **(C)** Insoluble fraction of Aβ in the prefrontal cortex. WT *N* = 6, APP/PS1 *N* = 6, APP/PS1 + *A. muciniphila N* = 6, APP/PS1 + *A. muciniphila* + GOS *N* = 8. Data are presented as mean ± SEM. ^*^p ≤ 0.05 and ^***^p ≤ 0.001.

The levels of soluble and insoluble Aβ_(1–42)_ monomers in extracted hippocampus and prefrontal cortex were measured after 7 months of *A. muciniphila* supplementation. As expected, APP/PS1 animals had significantly higher amount of Aβ_(1–42)_ in both soluble and insoluble fractions (Figures 5A,B). Supplementation with either *A. muciniphila* or combination of *A. muciniphila* and GOS had no effect on Aβ_(1–42)_ burden in the hippocampus. In the prefrontal cortex, Aβ (1–42) burden did not differ significantly between groups, with no notable reduction observed following *A. muciniphila* supplementation alone or in combination with GOS (Figure 5C).

### Changes in the gut microbiota due to the administration of *Akkermansia muciniphila* and GOS

**Figure 6:**
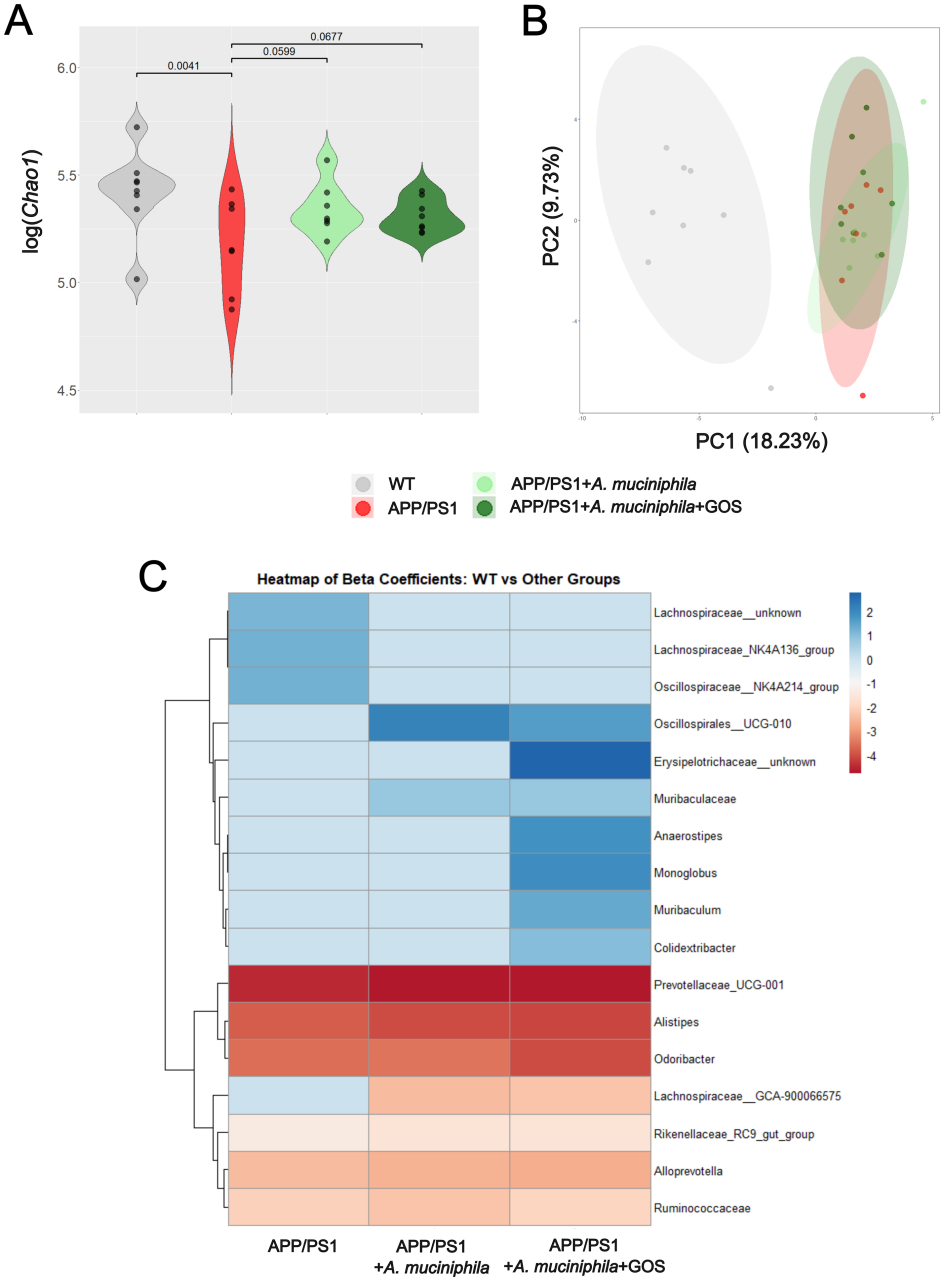
Composition of the gut microbiota in WT and APP/PS1 animals after 7 months of treatment. **(A)** Violin plot of *Chao1* diversity between study groups. **(B)** PCA plot illustrating group differences in PC 1 and 2. **(C)** Heatmap of differential abundant taxa *β* coefficients differences. WT versus APP variants. WT, wild type; APP, APP/PS1; APP A, APP/PS1 + *A. muciniphila*; APP A G, APP/PS1 + *A. muciniphila* + GOS. Associations assessed through linear regression, adjusting for whole gut transit time. Results with *p* < 0.05 were considered significant and reported in the plot. The association between specific taxa, at genus level, and study groups was assessed through general linear models, adjusting for whole gut transit time. Reported taxa where those with Benjamini–Hochberg adjusted *p* ≤ 0.05.

A total of 2,500 ASVs were identified in 30 samples based on raw counts. After aggregation at the genus level, 67 taxa were identified in the dataset. We found that the *Chao1* alpha diversity index was significantly higher in the WT group compared to the APP/PS1 group (*p* = 0.004) (Figure 6A, Supplementary Table S1). However, for the calculated alpha diversity indices, *Shannon* and *Simpson*, we found no significant differences between groups (Supplementary Tables S2, S3).

The PCA plot in Figure 6B shows that the top two axes, PC1 and PC2, account for 18.23 and 9.73% of the total variation, respectively. The results of the PERMANOVA indicated that the group variable significantly influenced the composition of the gut microbiota, with 25% of the variance explained by differences between the groups. This suggests that group membership was an important factor influencing the gut microbiota. The *F*-value of 3.075 and the *p*-value of 0.001 indicated that these differences were highly statistically significant, suggesting that supplementation contributed to the observed group differences (Table 2).

**Table 2:** Summary of PERMANOVA analysis for gut microbiota community composition by study group.

	Df	Sum of sqs	*R* ^2^	*F*	Pr(>*F*)
Group	3	809.700	0.249	3.075	0.001
Gut transit duration (s)	1	114.200	0.035	1.301	0.156
Residual	25	2194.600	0.674		
Total	29	3255.400	1.000		

The model was adjusted for whole gut transit time and *p* < 0.05 as significant threshold.

The results of the differential abundance analysis are summarized in Figure 6C and Supplementary Table S4. In summary, we identified a total of nine genera associated with APP/PS1 compared to WT (three positively associated), 10 genera associated with APP/PS1 + *A. muciniphila* compared to WT (three positively associated), and 14 genera associated with APP/PS1 + *A. muciniphila* + GOS compared to WT (seven positively associated). The abundance of an unknown genus from the Erysipelotrichaceae family was positively associated with APP/PS1 + *A. muciniphila* + GOS compared to APP/PS1 (*β* = 2.338, *p* = 0.043).

**Figure 7:**
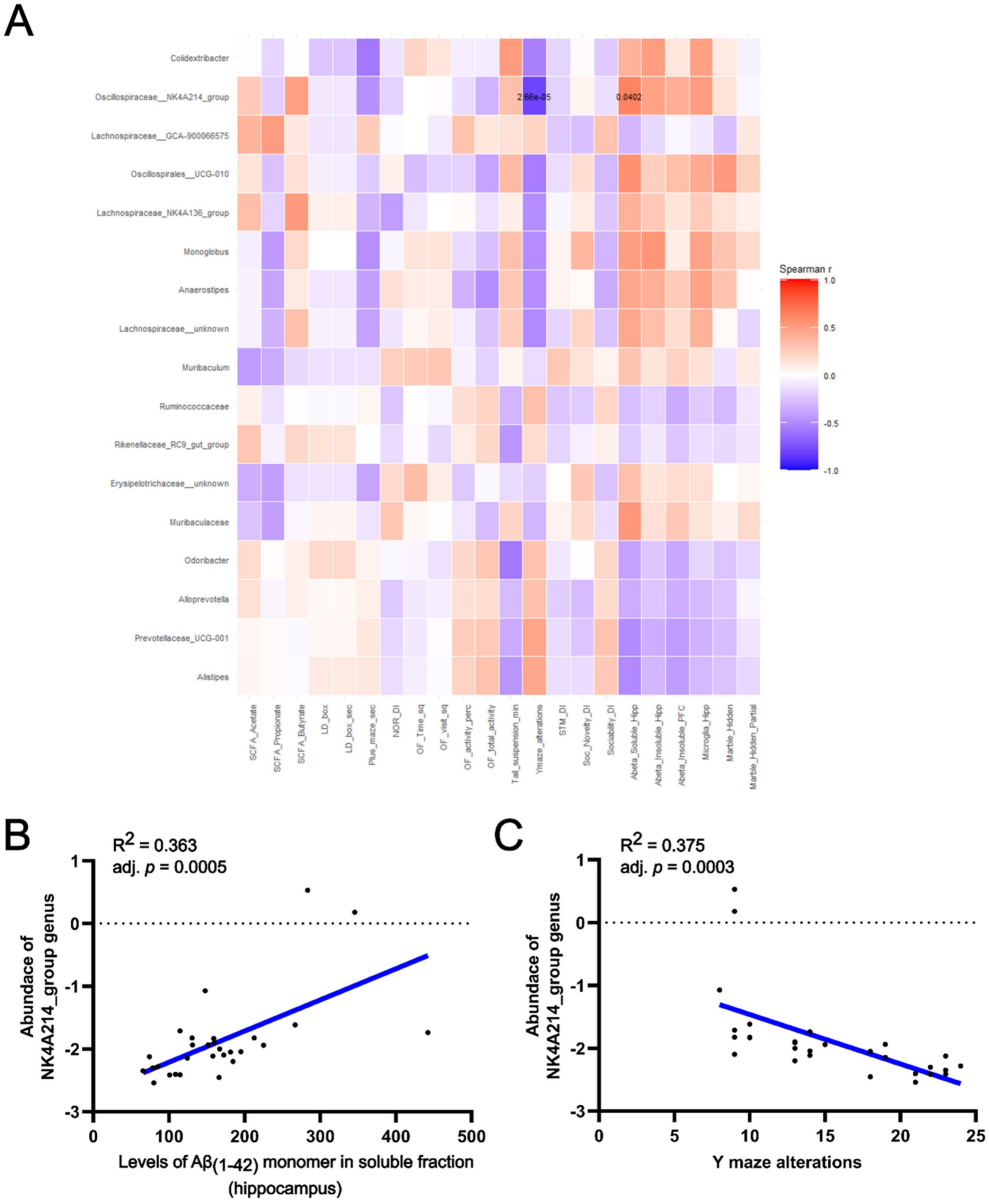
The correlation analysis between the detected bacterial genera and various analyzed variables in the study. **(A)** Heatmap showing the Spearman’s correlation between differential abundant taxa and different variables of interest. Each cell of the grid represents the correlation between a pair of variables, colors indicate the strength and direction of the correlation, darker or lighter shades reflect the magnitude of the correlation. Significant correlations are indicated by *p*-values in the corresponding heatmap cells. Correlations between the genus NK4A214 group from *Oscillospiraceae* family. **(B)** Level of soluble Aβ_(1–42)_ monomers in the hippocampus. **(C)** Number of alterations animals made in the Y-maze test.

The correlation analysis between the detected bacterial genera and the obtained data variables revealed two significant correlations, both involving the genus NK4A214 group from the *Oscillospiraceae* family (Figure 7A). We found a moderate positive correlation (*r*_s_ = 0.626, adjusted *p* = 0.00002) between the abundance of the NK4A214 group and the soluble fraction of Aβ_(1–42)_ monomer in the hippocampus (Figure 7B). Furthermore, there was a strong negative correlation (*r*_s_ = −0.807, adjusted *p* = 0,040) between the same NK4A214 group genus and the number of alterations performed by mice during the Y-maze test (Figure 7C). Taken together, these results suggest a potential role of the NK4A214 group genus in the development of AD. Similar results were observed with genus UCG-010 from the *Oscillospirales* family (Figure 7A). Here, we observed a trend that UCG-010 abundance correlated positively (*r*_s_ = 0.564, adjusted *p* = 0.079) with the levels of Aβ_(1–42)_ monomer in the hippocampus and a trend that UCG-010 abundance negatively correlated (*r*_s_ = −0.560, adjusted *p* = 0.079) with the number of alterations performed in the Y-maze test (Supplementary Table S5).

We found no significant correlation between the study groups and the abundance of the *Akkermansia* genus or *A. muciniphila* (Supplementary Tables S6, S7). The lack of an increase in *A. muciniphila* abundance in the gut, even after long-term supplementation, suggests that higher colonization of *A. muciniphila* is not essential to exert a significant impact on the host microbial community.

### Regulation of SCFA in the gut by the supplementation of *Akkermansia muciniphila* and GOS

**Figure 8:**
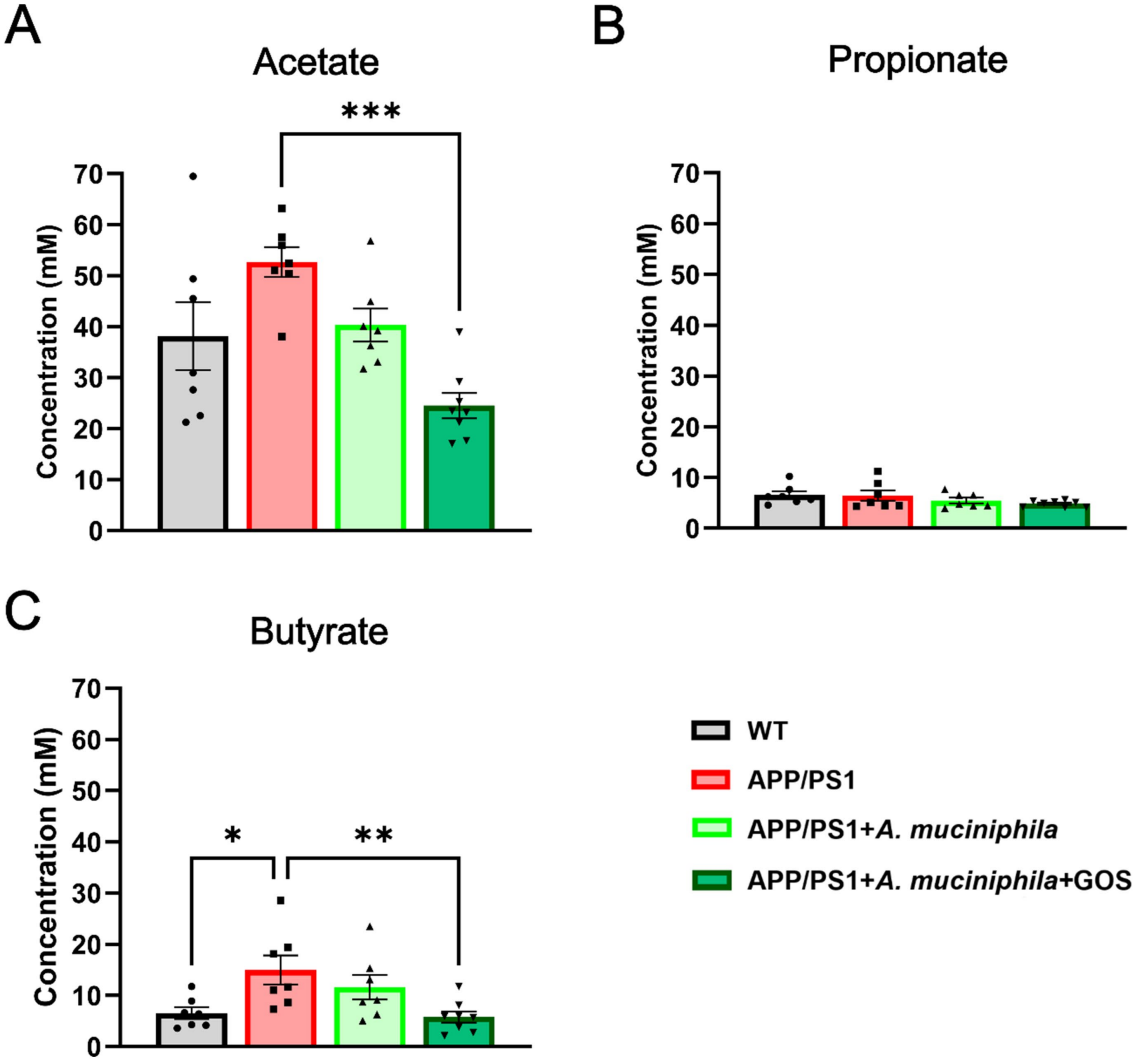
Gas chromatography of SCFA. **(A)** Concentration of acetate in the cecum. **(B)** Concentration of propionate in the cecum. **(C)** Concentration of butyrate in the cecum. WT *N* = 8, APP/PS1 *N* = 7, APP/PS1 + *Akkermansia N* = 7, APP/PS1 + *Akkermansia* + GOS *N* = 8. Data are presented as mean ± SEM. ^*^p ≤ 0.05, ^**^p ≤ 0.01, and ^***^p ≤ 0.001. Differences are shown compared to the APP/PS1 group.

The changes in the gut microbiota observed after the administration of *A. muciniphila* indicate changes in the metabolic activity of the bacterial community. The contents of the cecum were analyzed by gas chromatography and the concentrations of acetate, propionate, and butyrate were determined. We observed a trend (*p* = 0.052) towards increased acetate concentrations in APP/PS1 animals, which was significantly reduced by the administration of *A. muciniphila* and GOS (Figure 8A). In contrast, we observed no differences in concentration of propionate (Figure 8B). The changes in SCFA concentrations were particularly striking for butyrate, where APP/PS1 animals had significantly higher levels, and the administration of *A. muciniphila* and GOS brought these levels back to those of the WT controls (Figure 8C). These results indicate that long-term supplementation of *A. muciniphila* and GOS modulates the concentration of SCFA in the APP/PS1 AD model to WT levels.

### Modulation of hippocampal microglial percentage by supplementation with *Akkermansia muciniphila* and GOS

**Figure 9:**
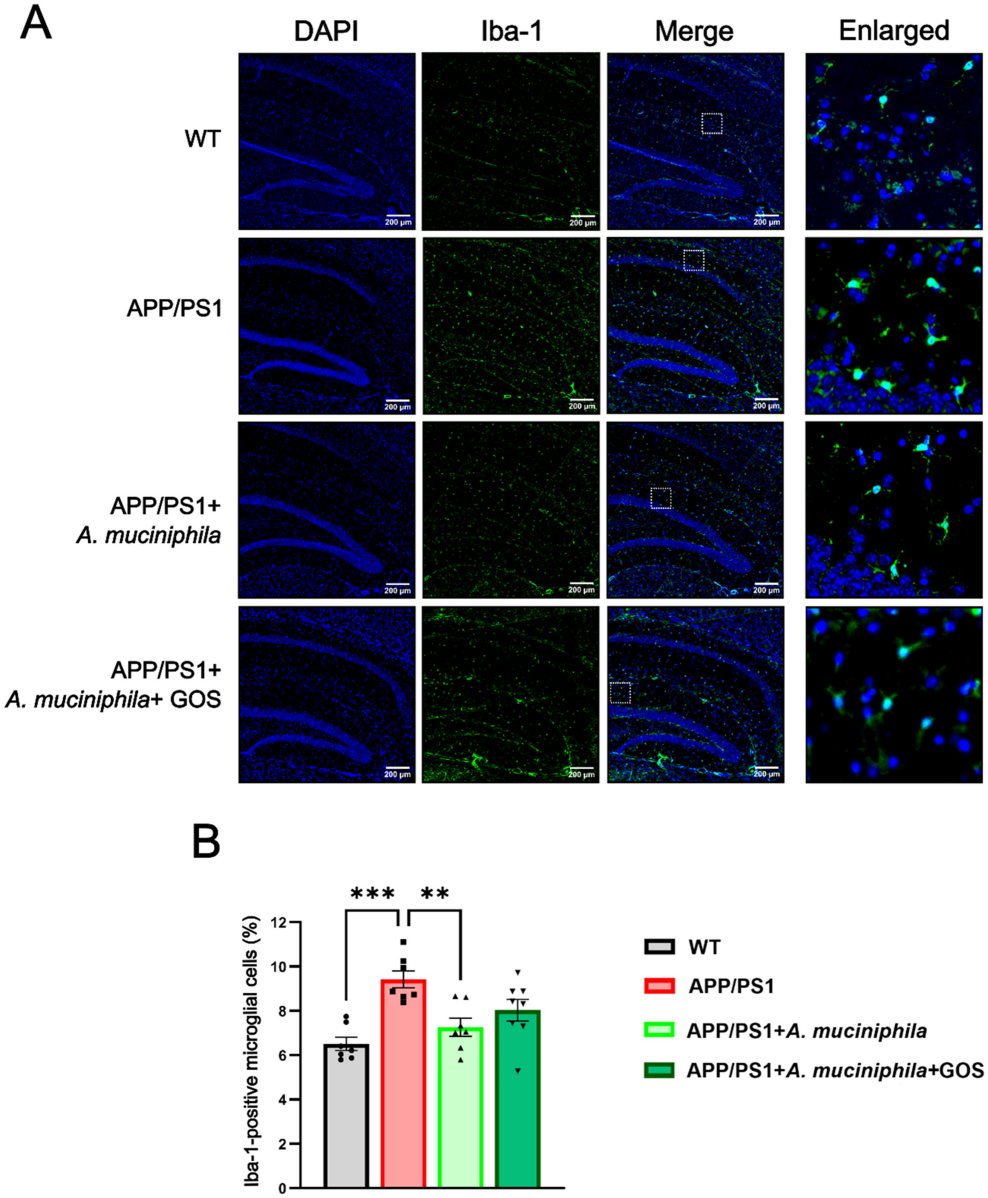
Immunohistochemical analysis of microglia in hippocampus. DAPI—blue, Iba1—green. DAPI - 4’,6-diamidino-2-phenylindole. Iba-1 - Ionized calcium-binding adaptor molecule 1. WT *N* = 7, APP/PS1 *N* = 7, APP/PS1 + *A. muciniphila N* = 6, APP/ PS1 + *A. muciniphila* + GOS *N* = 8. Data are presented as mean ± SEM. ^**^p ≤ 0.01 and ^***^p ≤ 0.001. Differences are shown compared to the APP/PS1 group.

Immunohistochemical analysis of the microglia marker Iba1 was performed on hippocampal slices (Figure 9). We observed a significantly increased percentage of microglia in the hippocampus of APP/PS1 animals, indicating a higher level of inflammation. Supplementation with *A. muciniphila* significantly prevented this increase and partially restored the phenotype to the WT levels. The addition of GOS in addition to the *A. muciniphila* treatment diminished the positive effect of the probiotic. These results suggest that supplementation with *A. muciniphila* alone is able to prevent pathological increase in microglia abundance in the hippocampus of APP/PS1 animals.

## Discussion

In this study, we tested the therapeutic potential of *A. muciniphila* in the treatment of AD symptoms. For this purpose, we intermittently administered the probiotic bacteria *A. muciniphila* to the APP/PS1 mouse line three times a week for 7 months. Additionally, we investigated whether prebiotic GOS has a synergistic effect with *A. muciniphila*. This naturally gut residing bacteria has a promising clinical application and has been described as a next-generation probiotic (Al-Fakhrany and Elekhnawy, 2024). *A. muciniphila* has been shown to be safe and to provide multiple health benefits, including strengthening gut barrier integrity, improving the metabolic profile of the host, and reducing inflammation (Li et al., 2024). To quantify and evaluate the effect of these interventions, we measured physiological parameters, including body weight, intestinal transit time, fasting glucose levels, glucose metabolism, multiple behavior traits, gut microbiota composition and diversity, levels of SCFA in the cecum, burden of Aβ_(1–42)_ monomers in the hippocampus and prefrontal cortex, and number of microglia cells in the brain.

In addition to Aβ accumulation, a number of studies have reported that APP/PS1 mice exhibit behavioral deficits in various psychological parameters (Ferguson et al., 2013; Sasaguri et al., 2017). APP/PS1 animals show no changes in body weight or fasting glucose levels at 6–7 months of age compared to WT mice (Walker et al., 2017). In our study, 10-month-old APP/PS1 mice exhibited no differences in body weight, but a significant increase in fasting glucose, which was completely reversed by the administration of *A. muciniphila* and GOS. Furthermore, APP/PS1 exhibited impaired glucose metabolism, which was normalized to WT levels by administration of the symbiotic. Metabolic disorders, including impaired glucose metabolism, can modulate the immune response and contribute to elevated levels of neuroinflammation (Lee et al., 2024). Therefore, improving glucose metabolism by administering *A. muciniphila* and GOS could be a useful adjunct to conventional therapies for AD.

AD is associated with colonic dysfunction and an increased incidence of gastrointestinal symptoms. Although human studies report constipation, decreased intestinal motility, and prolonged transit time (Kang J. et al., 2024), studies using AD mouse models have observed a progressive decrease in intestinal transit time that progresses with age (Stoye et al., 2020). We found a significantly shorter intestinal transit time in APP/PS1 mice compared to WT mice, while supplementation with *A. muciniphila* and GOS protected against this decline. *A. muciniphila* and GOS exert their effects locally on the gastrointestinal system and systemically on the metabolic health of the host.

APP/PS1 is a preclinical AD-like model that is used extensively in studies to screen new drugs and closely mimics AD-like phenotype. These animals have anxiety, memory deficits, hyperactivity, and social deficits (Huang et al., 2016). The functional significance of *A. muciniphila* and GOS supplementation can be seen in behavioral experiments. We observed an increase in both the number of visits and time spent in the center of the open field test, indicating lower levels of anxiety following the administration of tested prebiotics and probiotics. Our data support the findings of Guo et al. (2024), who showed that *A. muciniphila* had a mild but significant effect on attenuating alcohol-induced depression in mice. Although compulsive behavior is not a hallmark of AD, APP/PS1 animals are known to exhibit increased marble burying (Peng et al., 2021). We observed a trend towards a 2-fold increase (*p* = 0.0592) in the number of buried marbles by APP/PS1 animals compared to WT. In contrast, the group supplemented with *A. muciniphila* and GOS showed a trend (*p* = 0.0581) towards fewer buried marbles, suggesting less impulsive or anxiety-induced behavior. On the other hand, memory and social deficits are among the first symptoms reported by AD patients (Zhu et al., 2017). We observed a partial but significant recovery of spatial memory in the Y-maze test and an improvement in declarative long-term memory. These results are consistent with those of Kang E. J. et al. (2024), who demonstrated the ability of *A. muciniphila* to improve memory in a mouse model of liver disease.

The amount, solubility and location of Aβ deposits are considered to be the main factors in the pathophysiology and symptoms of AD (Murphy and LeVine, 2010). We observed no effect of *A. muciniphila* and GOS supplementation on soluble and insoluble fractions Aβ_(1–42)_ monomers in the hippocampus. These results suggest that the synbiotic exerts its effects on the CNS and behavior through an Aβ-independent mechanism, potentially via the regulation of neuroinflammation or tau accumulation. Detailed investigation into neuroinflammation markers, such as TNF-α, interleukins and chemokines could provide valuable mechanisms to the effects of *A. muciniphila* and GOS.

Metabolites derived from the gut microbiota have been reported to be key regulators of the immune system and contribute to the development of various neuropsychiatric disorders. The most abundant gut-derived metabolites are known to have neuroactive properties that alter histone deacetylation and the BDNF expression (Erny et al., 2015). We found that APP/PS1 mice had elevated acetate and butyrate concentrations in the cecum, which is in contrast to the findings Zhang et al. (2017) who showed decreased SCFA concentrations in the feces (Yilmaz et al., 2017). However, several studies reported increased SCFA concentrations in the saliva of AD patients (Figueira et al., 2016; Yilmaz et al., 2017). Our data indicate that long-term supplementation with *A. muciniphila* and GOS significantly modulates acetate and butyrate levels in the cecum, with butyrate levels being restored to WT levels. The regulated butyrate level could be one of the mechanisms underlying the observed improvements in memory and reduced anxiety, as butyrate is known to inhibit histone deacetylation in the hippocampus (Fernando et al., 2020; Topuz et al., 2020). Studies utilizing germ-free mice supports the idea of SCFAs contributing to the AD phenotype as exposure to SCFAs increases apolipoprotein E expression in microglia, inducing faster Aβ deposition during early amyloidogenesis (Colombo et al., 2021).

There is ample evidence that the gut microbiota of AD patients and animal models is imbalanced and has reduced diversity, which contributes to the progression of AD (Zhang et al., 2017; Li et al., 2019; Megur et al., 2020; Qian et al., 2021). Our *16S* rRNA sequencing data confirmed the lower diversity in the AD model and showed a significantly lower *Chao1* index in the APP/PS1 group compared to the WT group. Supplementation with *A. muciniphila* or *A. muciniphila* and GOS had a promising trend of a positive effect (*p* = 0.0599 and *p* = 0.0677, respectively) on the *Chao1* index, representing improved alpha diversity of the gut microbiota. Beta diversity analysis confirmed the efficacy of supplementation with *A. muciniphila* and GOS, with 25% of the variance between groups, explained by the supplementation. These results are similar to other interventional studies with *A. muciniphila* showing increased diversity of the gut microbiota (Khalili et al., 2024). A more detailed analysis of specific bacterial differences revealed that *A. muciniphila* and GOS supplementation significantly enriched the *Erysipelotrichaceae* family, which are known to produce SCFA, particularly butyrate (Estaki et al., 2016). However, the significance of *Erysipelotrichaceae* abundance in the context of AD is still unclear. These results suggest that the observed increase in acetate and butyrate in APP/PS1 animals may not be directly caused by bacterial species differences but by the shift in metabolic activity of the existing microbial community.

The correlation analysis highlighted the NK4A214 group genus from the *Oscillospiraceae* family as a potentially important bacterial taxa in the early stages of AD. We found a positive association with the amount of soluble Aβ_(1–42)_ in the hippocampus and a negative association with the number of alterations in the Y-maze, a test of spatial memory. Aβ deposits have been shown to damage the hippocampus and lead to cognitive impairment (Stepanichev et al., 2004; Stevens et al., 2022), suggesting a possible link between the abundance of NK4A214 group bacteria in the gut and the AD. To the best of the our knowledge, the NK4A214 group genus has not previously been associated with AD, although it was associated with lower bone mineral density and obesity (Burakova et al., 2022; Simpson et al., 2024). Obesity is an important risk factor of AD and other dementias and has been linked to cognitive deficits and neuroinflammation (Miller and Spencer, 2014; Tsai et al., 2019; Flores-Cordero et al., 2022). Furthermore, bacteria from the *Oscillospiraceae* family have previously been associated with mild cognitive impairment observed in AD (Park and Wu, 2022). Additionally, *UCG-005* genus from the *Oscillospiraceae* family showed a significant association with Aβ levels in AD patients (Verhaar et al., 2022). These results provide the first preliminary evidence for the importance of the abundance of the NK4A214 group genus and *Oscillospiraceae* family in the development of AD and emphasize the potential significance of strategies aimed at modulating *Oscillospiraceae* levels in AD patients. Although we acknowledge that a deeper species-or strain-level profiling using shotgun metagenomic analysis could help better identify microbial units contributing to AD phenotype.

Neuroinflammation plays an important role in the pathophysiology and development of AD and is frequently observed in AD animal models (López-González et al., 2015; Heneka et al., 2024). In our study, APP/PS1 mice exhibited a significantly higher proportion of microglial cells in the hippocampus compared to WT animals, confirming the findings of Holloway et al. (2020). *A. muciniphila* supplementation has previously been described to reduce neuroinflammation by decreasing proinflammatory IL-6 and increasing anti-inflammatory IL-10 (Yilmaz et al., 2017). Additionally, supplementation with GOS reduce the expression of TNF-α and increase the expression of IL-10 supporting the potential for synergistic anti-inflammatory effect of *A. muciniphila* and GOS (Vijaya et al., 2024). Therefore, it is likely that part of the observed effect on microglia may be mediated by altered cytokine signaling, although this was not directly tested in the present study. Furthermore, Laffer et al. (2019) reported that IL-10 prevents microglial activation, providing a potential explanation for our findings that *A. muciniphila* supplementation reduced the proportion of microglial cells in the hippocampus of APP/PS1 mice. Regulation of microglia abundance could be facilitated through reduction in SCFAs, which have been shown to activate toll-like receptors which promote abnormal activation and proliferation of microglia (Lin et al., 2015; Miao et al., 2023). However, we did not observe a synergistic effect with the addition of GOS in regulating microglia abundance. These results are the first steps in highlighting the potential of *A. muciniphila* supplementation in reducing neuroinflammation. However, further studies are required to comprehensively investigate microglial markers and interleukin levels in the brain to confirm these findings.

The results of this study are consistent with those of a similar study by Ou et al. (2020), in which APP/PS1 animals were fed a high-fat diet and gavaged with *A. muciniphila* daily for six months. Both our study and that of Ou showed that the administration of *A. muciniphila* reduce levels of fasting glucose and improve behavior. In contrast to Ou’s et al. (2020) findings, we did not observe any effect of *A. muciniphila* supplementation on Aβ load. These differences could be due to the fact that we did not use a high-fat diet, which exacerbates the progression of AD. In addition, we administered *A. muciniphila* less frequently (three times a week instead of everyday) and have not detected the increase of *A. muciniphila* after the supplementation. Due to the absence of longitudinal gut microbiota analysis, we were unable to determine whether *A. muciniphila* failed to colonize the gut or was successfully established but subsequently eliminated during the 7-month supplementation period. Use of antibiotics before the administration could reduce the abundance of residing gut bacteria, improve colonization efficiency, and should be explored in future studies. It can therefore be assumed that the observed positive effects of the supplementation were not directly mediated by gut-residing *A. muciniphila*. Nevertheless, our results suggest that the administration three times per week is sufficient to achieve beneficial effects on microbiota diversity, glucose metabolism, overabundance of microglia, and to alleviate the behavioral changes associated with AD.

## Conclusion

In summary, 7 months intermittent supplementation with *A. muciniphila* and GOS had a broad beneficial effect on the APP/PS1 AD mouse model. These included normalizing glucose metabolism and intestinal transit time, reducing anxiety, improving memory, partially restoring activity levels, regulating cecal SCFA levels, and improving the richness of the gut microbiota with no detectable adverse effects. The prebiotic GOS enhanced the efficacy of supplementation with *A. muciniphila*, although *A. muciniphila* alone was sufficient to increase the diversity of the gut microbiota and reduce the proportion of microglia in the hippocampus of APP/PS1 animals. Furthermore, we highlighted the NK4A214 group genus as a bacterial taxa contributing to Aβ_(1–42)_ deposition and memory deficits. Overall, *A. muciniphila* and GOS supplementation demonstrate the potential of gut microbiota-based treatment strategies in the management of AD symptoms in an Aβ-independent matter.

## Data Availability

The original contributions presented in the study are publicly available. This data can be found here: https://doi.org/10.6084/m9.figshare.29291003.v1.
