# Supplementary material for: Intermittent supplementation with Akkermansia muciniphila and galactooligosaccharides modulates Alzheimer’s disease progression, gut microbiota, and colon short-chain fatty acid profiles in mice
Source: Front Aging Neurosci. 2025 Jun 30;17:1617980. doi: 10.3389/fnagi.2025.1617980 (PMC12256538; doi:10.3389/fnagi.2025.1617980)
Supplement: Supplementary file 1 [file Data_Sheet_1.docx]

Supplementary Material

**Supplementary Table S1.** Summary of linear regression model for *Chao1* diversity index across study groups.

|  | **Ref. Group** | **Estimate** | **Std. Error** | **t value** | **Pr(>\|t\|)** |
| --- | --- | --- | --- | --- | --- |
| (Intercept) | WT | 285.799 | 43.380 | 6.588 | 6.67E-07 |
| Group: APP_A | WT | -31.104 | 18.896 | -1.646 | 0.112 |
| Group: APP | WT | -65.519 | 20.735 | -3.160 | 0.004 |
| Group: APP_A_G | WT | -31.215 | 16.344 | -1.910 | 0.068 |
| (Intercept) | APP | 220.280 | 31.823 | 6.922 | 2.96E-07 |
| Group: APP_A | APP | 34.415 | 17.460 | 1.971 | 0.060 |
| Group: APP_A_G | APP | 34.304 | 18.974 | 1.808 | 0.083 |
| (Intercept) | APP_A | 254.694 | 34.982 | 7.281 | 1.25E-07 |
| Group: APP_A_G | APP_A | -0.110 | 17.578 | -0.006 | 0.995 |

WT, wild type; APP, APP/PS1; APP A, APP/PS1 + *A. muciniphila*; APP A G, APP/PS1 + *A. muciniphila* + GOS. Associations assessed through linear regression, adjusting for Carmine intestinal transit time. Results with *P* < 0.05 were considered significant.

**Supplementary Table S2**. Summary of linear regression model for *Shannon* diversity index across study groups.

|  | **Ref. Group** | **Estimate** | **Std. Error** | **t value** | **Pr(>\|t\|)** |
| --- | --- | --- | --- | --- | --- |
| (Intercept) | WT | 5.163 | 0.413 | 12.505 | 2.96E-12 |
| Group: APP_A | WT | -0.043 | 0.180 | -0.239 | 0.813 |
| Group: APP | WT | -0.354 | 0.197 | -1.795 | 0.085 |
| Group: APP_A_G | WT | -0.007 | 0.156 | -0.045 | 0.965 |
| (Intercept) | APP | 4.809 | 0.303 | 15.876 | 1.44E-14 |
| Group: APP_A | APP | 0.311 | 0.166 | 1.873 | 0.073 |
| Group: APP_A_G | APP | 0.347 | 0.181 | 1.923 | 0.066 |
| (Intercept) | APP_A | 5.120 | 0.333 | 15.377 | 2.99E-14 |
| Group: APP_A_G | APP_A | 0.036 | 0.167 | 0.216 | 0.831 |

WT, wild type; APP, APP/PS1; APP A, APP/PS1 + *A. muciniphila*; APP A G, APP/PS1 + *A. muciniphila* + GOS. Associations assessed through linear regression, adjusting for Carmine intestinal transit time. Results with *P* < 0.05 were considered significant.

**Supplementary Table S3**. Summary of linear regression model for *Simpson* diversity index across study groups.

|  | **Ref. Group** | **Estimate** | **Std. Error** | **t value** | **Pr(>\|t\|)** |
| --- | --- | --- | --- | --- | --- |
| (Intercept) | WT | 0.989 | 0.015 | 65.452 | <2e-16 |
| Group: APP_A | WT | 0.005 | 0.007 | 0.722 | 0.477 |
| Group: APP | WT | -0.004 | 0.007 | -0.554 | 0.585 |
| Group: APP_A_G | WT | 0.005 | 0.006 | 0.963 | 0.345 |
| (Intercept) | APP | 0.985 | 0.011 | 88.860 | <2e-16 |
| Group: APP_A | APP | 0.009 | 0.006 | 1.439 | 0.163 |
| Group: APP_A_G | APP | 0.009 | 0.007 | 1.434 | 0.164 |
| (Intercept) | APP_A | 0.994 | 0.012 | 81.555 | <2e-16 |
| Group: APP_A_G | APP_A | 0.001 | 0.006 | 0.119 | 0.906 |

WT, wild type; APP, APP/PS1; APP A, APP/PS1 + *A. muciniphila*; APP A G, APP/PS1 + *A. muciniphila* + GOS. Associations assessed through linear regression, adjusting for Carmine intestinal transit time. Results with *P* < 0.05 were considered significant.

**Supplementary Table S4**. Summary of differential abundant taxa associated with Groups APP, APP A, APP A G versus WT.

| **Taxa** | **Ref** | **Group** | **β coef** | **Std err** | ***P*** | **Adj *P*** |
| --- | --- | --- | --- | --- | --- | --- |
| *Alistipes* | WT | APP | -3.863 | 0.152 | 2.36E-19 | 9.21E-18 |
| *Prevotellaceae_UCG.001* | WT | APP | -4.529 | 0.200 | 3.38E-18 | 1.10E-16 |
| *Alloprevotella* | WT | APP | -2.434 | 0.431 | 7.00E-06 | 1.37E-04 |
| *Odoribacter* | WT | APP | -3.593 | 0.704 | 2.83E-05 | 4.60E-04 |
| *Lachnospiraceae_unknown* | WT | APP | 1.144 | 0.293 | 6.32E-04 | 5.87E-03 |
| *Lachnospiraceae_NK4A136_*group | WT | APP | 1.235 | 0.336 | 1.14E-03 | 9.26E-03 |
| *Ruminococcaceae* | WT | APP | -2.125 | 0.592 | 1.41E-03 | 1.10E-02 |
| *NK4A214_group* | WT | APP | 1.236 | 0.390 | 4.04E-03 | 2.81E-02 |
| *Rikenellaceae_RC9_*gut_group | WT | APP | -1.430 | 0.460 | 4.66E-03 | 3.03E-02 |
| *Alistipes* | WT | APP_A | -4.053 | 0.139 | 7.77E-21 | 5.05E-19 |
| *Prevotellaceae_UCG.001* | WT | APP_A | -4.719 | 0.182 | 1.35E-19 | 6.58E-18 |
| *Alloprevotella* | WT | APP_A | -2.624 | 0.393 | 5.26E-07 | 1.14E-05 |
| *Odoribacter* | WT | APP_A | -3.530 | 0.641 | 1.02E-05 | 1.80E-04 |
| *Ruminococcaceae* | WT | APP_A | -2.352 | 0.540 | 1.97E-04 | 2.26E-03 |
| *Muribaculaceae* | WT | APP_A | 0.808 | 0.187 | 2.14E-04 | 2.31E-03 |
| *Rikenellaceae_RC9_*gut_group | WT | APP_A | -1.620 | 0.419 | 7.03E-04 | 6.23E-03 |
| *UCG.010* | WT | APP_A | 2.141 | 0.629 | 2.23E-03 | 1.67E-02 |
| *Chloroplast* | WT | APP_A | 2.298 | 0.760 | 5.69E-03 | 3.58E-02 |
| *GCA.900066575* | WT | APP_A | -2.488 | 0.832 | 6.16E-03 | 3.75E-02 |
| *Alistipes* | WT | APP_A_G | -4.104 | 0.120 | 1.66E-22 | 3.24E-20 |
| *Prevotellaceae_UCG.001* | WT | APP_A_G | -4.770 | 0.157 | 3.09E-21 | 3.01E-19 |
| *Alloprevotella* | WT | APP_A_G | -2.675 | 0.340 | 3.11E-08 | 8.66E-07 |
| *Odoribacter* | WT | APP_A_G | -4.016 | 0.555 | 1.38E-07 | 3.36E-06 |
| *Muribaculaceae* | WT | APP_A_G | 0.795 | 0.162 | 4.53E-05 | 6.79E-04 |
| *Erysipelotrichaceae_unknown* | WT | APP_A_G | 2.777 | 0.576 | 5.86E-05 | 8.16E-04 |
| *Rikenellaceae_RC9_*gut_group | WT | APP_A_G | -1.671 | 0.363 | 1.04E-04 | 1.35E-03 |
| *Ruminococcaceae* | WT | APP_A_G | -2.044 | 0.467 | 1.86E-04 | 2.26E-03 |
| *Muribaculum* | WT | APP_A_G | 1.404 | 0.327 | 2.29E-04 | 2.35E-03 |
| *Anaerostipes* | WT | APP_A_G | 1.850 | 0.473 | 6.19E-04 | 5.87E-03 |
| *Monoglobus* | WT | APP_A_G | 1.925 | 0.518 | 1.02E-03 | 8.67E-03 |
| *GCA.900066575* | WT | APP_A_G | -2.325 | 0.719 | 3.44E-03 | 2.48E-02 |
| *Colidextribacter* | WT | APP_A_G | 1.026 | 0.326 | 4.28E-03 | 2.87E-02 |
| *UCG.010* | WT | APP_A_G | 1.573 | 0.544 | 7.77E-03 | 4.59E-02 |

WT, wild type; APP, APP/PS1; APP A, APP/PS1 + *A. muciniphila*; APP A G, APP/PS1 + *A. muciniphila* + GOS. Associations tested with general linear models adjusting for Carmine intestinal transit time. Findings with Benjamini-Hochberg adjusted *P* < 0.05 were considered significant.

**Supplementary Table S5.** Results of Spearman’s correlation between differential abundance taxa and variables of interest. **The Excel spreadsheet “correlation.table.xlsx” can be accessed separately.**

**Supplementary Table S6**. Summary of linear regression model for *Akkermansia* genus abundance across study groups.

|  | **Ref. Group** | **Estimate** | **Std. Error** | **t value** | **Pr(>\|t\|)** |
| --- | --- | --- | --- | --- | --- |
| (Intercept) | WT | -0.434 | 1.306 | -0.332 | 0.742 |
| Group: APP_A | WT | -0.167 | 0.569 | -0.293 | 0.772 |
| Group: APP | WT | 0.306 | 0.624 | 0.49 | 0.629 |
| Group: APP_A_G | WT | -0.093 | 0.492 | -0.189 | 0.851 |
| (Intercept) | APP | -0.129 | 0.958 | -0.134 | 0.894 |
| Group: APP_A | APP | -0.472 | 0.526 | -0.898 | 0.378 |
| Group: APP_A_G | APP | -0.399 | 0.571 | -0.698 | 0.492 |
| (Intercept) | APP_A | -0.601 | 1.053 | -0.571 | 0.573 |
| Group: APP_A_G | APP_A | 0.074 | 0.529 | 0.139 | 0.891 |

WT, wild type; APP, APP/PS1; APP A, APP/PS1 + *A. muciniphila*; APP A G, APP/PS1 + *A. muciniphila* + GOS. Associations assessed through linear regression, adjusting for Carmine intestinal transit time. Results with *P* < 0.05 were considered significant.

**Supplementary Table S7**. Summary of linear regression model for *A. muciniphila* abundance across study groups.

|  | **Ref. Group** | **Estimate** | **Std. Error** | **t value** | **Pr(>\|t\|)** |
| --- | --- | --- | --- | --- | --- |
| (Intercept) | WT | -0.004 | 1.341 | -0.003 | 0.998 |
| Group: APP_A | WT | -0.252 | 0.584 | -0.432 | 0.669 |
| Group: APP | WT | 0.239 | 0.641 | 0.373 | 0.713 |
| Group: APP_A_G | WT | -0.169 | 0.505 | -0.335 | 0.74 |
| (Intercept) | APP | 0.235 | 0.984 | 0.239 | 0.813 |
| Group: APP_A | APP | -0.491 | 0.540 | -0.91 | 0.371 |
| Group: APP_A_G | APP | -0.408 | 0.586 | -0.696 | 0.493 |
| (Intercept) | APP_A | -0.256 | 1.081 | -0.237 | 0.815 |
| Group: APP_A_G | APP_A | 0.083 | 0.543 | 0.153 | 0.880 |

WT, wild type; APP, APP/PS1; APP A, APP/PS1 + *A. muciniphila*; APP A G, APP/PS1 + *A. muciniphila* + GOS. Associations assessed through linear regression, adjusting for Carmine intestinal transit time. Results with *P* < 0.05 were considered significant.


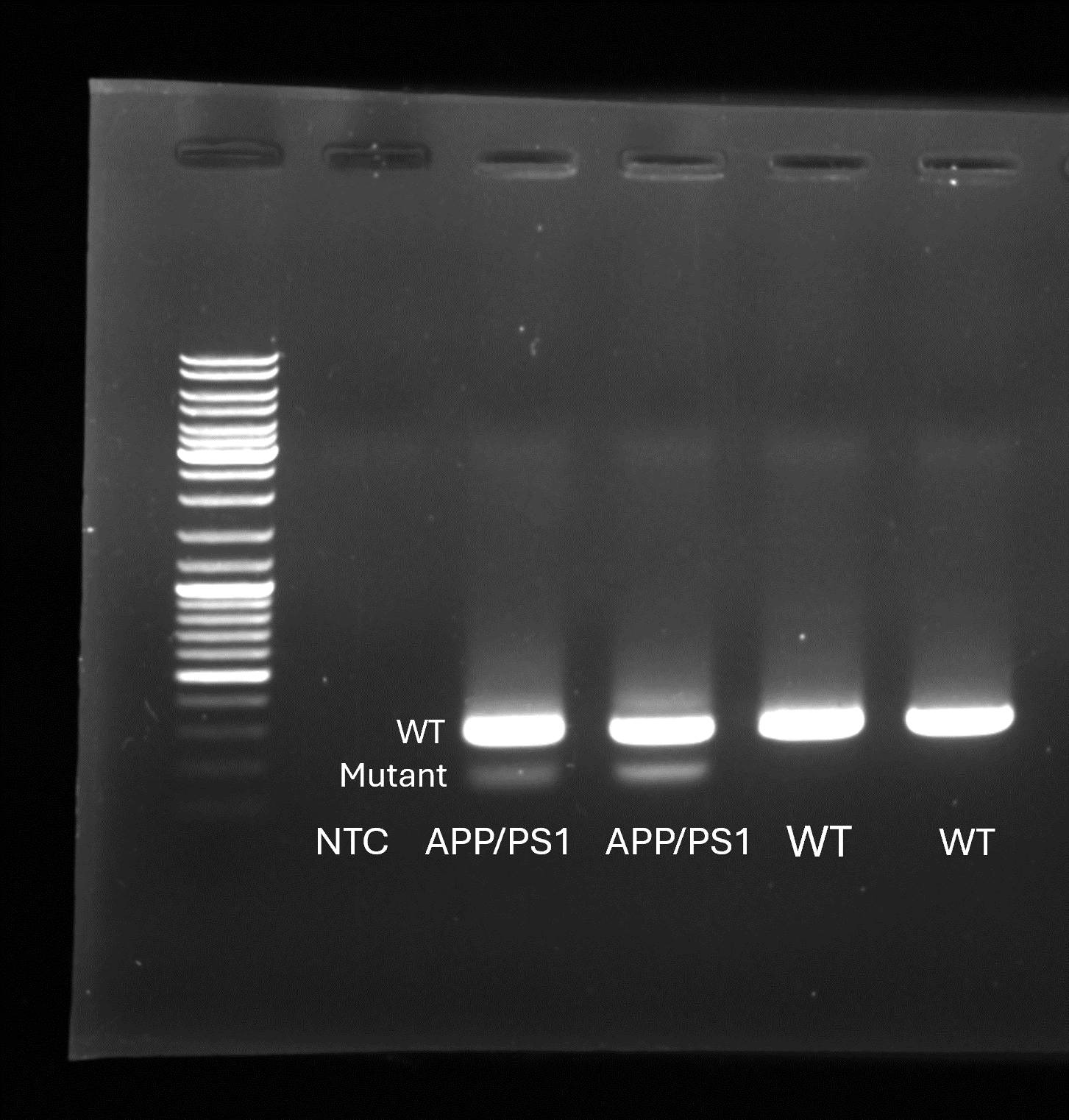


**Supplemental Figure S1.** Genotyping of APP/PS1 animals. WT fragment is 265 bp size and Mutant fragment is 142 bp.


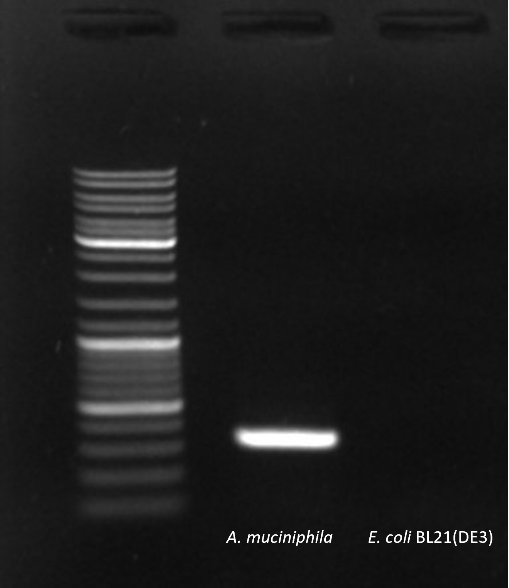


**Supplemental Figure S2.** Genotyping *A. muciniphila*. 1^st^ well - *A. muciniphila* administered in the experiment. 2^nd^ well – negative control (*E. coli*BL21(DE3)).


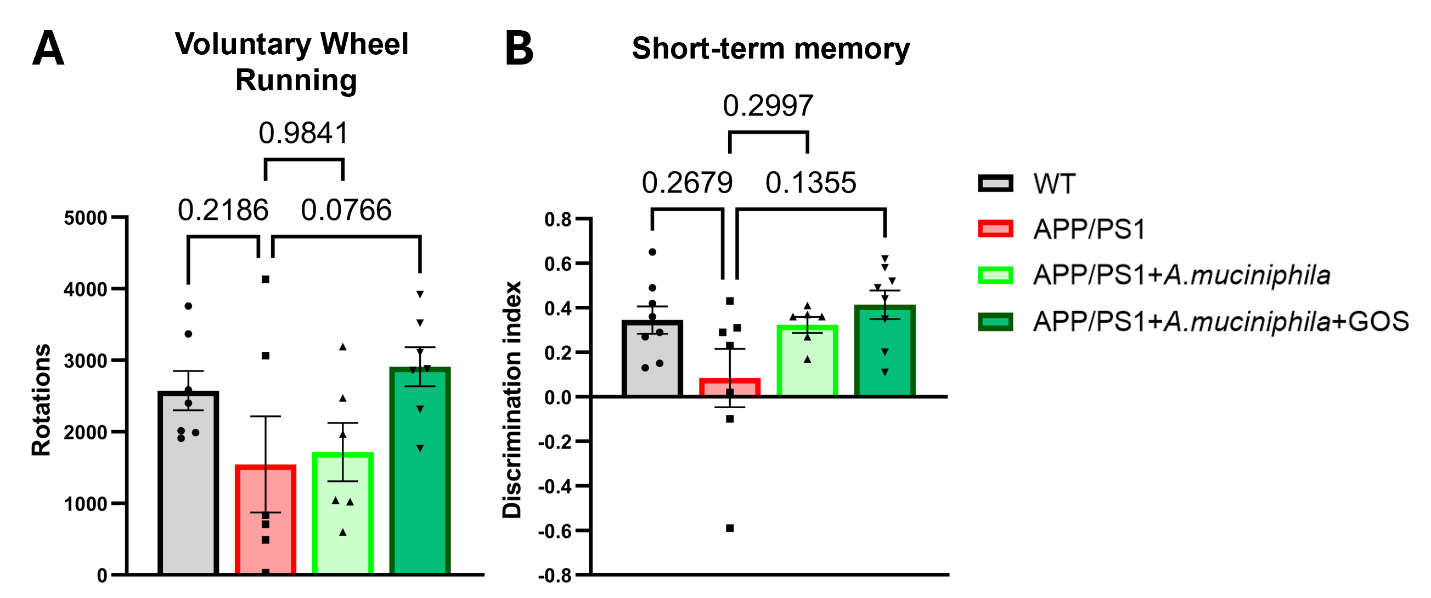


**Supplemental Figure S3.** Behavior assessment of APP/PS1 animals at 10 months of age. A – Activity measurement in voluntary wheel running test. B – Short-term memory test. WT N = 8, APP/PS1 N = 7, APP/PS1+ *A. muciniphila* N = 7, APP/PS1+ *A. muciniphila*+GOS N = 8. Data are presented as mean ± SEM.
